# Carrier Dynamics in Low-Dimensional and Wide-Bandgap Materials Probed by Terahertz Magneto-Optical Spectroscopy

**DOI:** 10.3390/nano16201298

**Published:** 2026-10-13

**Authors:** Ali Farooq, Muhammad Touqeer, Wen Xu, Muhammad Bilal, Huan Xiao, Muhammad Zeeshan, Muhammad Abid Anwar, Francois M. Peeters, Sichao Du

**Affiliations:** 1 Zhejiang Key Laboratory of Quantum Materials and Control, School of Information and Electrical Engineering, Hangzhou City University, Hangzhou 310015, China; alifarooq@mail.ustc.edu.cn; 2 State Key Laboratory of Silicon and Advanced Semiconductor Materials, School of Materials Science and Engineering, Zhejiang University, Hangzhou 310027, China; 3 Key Laboratory of Nanodevices and Applications, Suzhou Institute of Nano-Tech and Nano-Bionics, Chinese Academy of Sciences, Suzhou 215123, China; 4 Key Laboratory of Materials Physics, Institute of Solid State Physics, Hefei Institutes of Physical Science, Chinese Academy of Sciences, Hefei 230031, China; 5 School of Physics and Astronomy, Yunnan University, Kunming 650091, China; 6 School of Optoelectronic Science and Engineering, Anhui University, Hefei 230601, China; 7 Department of Physics, Chongqing Sanxia University of Science and Technology, Wanzhou 404100, China; 8 School of Nuclear Science and Technology, University of Science and Technology of China, Hefei 230026, China; 9 Jinhua Institute of Zhejiang University, Jinhua 321099, China; 10 Department of Physics, University of Antwerp, Groenenborgerlaan 171, B-2020 Antwerpen, Belgium; 11 Departamento de Física, Universidade Federal do Ceará, Fortaleza 60455-760, CE, Brazil; 12 Nanjing University of Information Science and Technology, Nanjing 210044, China; 13 College of Information Science and Electronic Engineering, Zhejiang University, Hangzhou 310027, China

**Keywords:** magneto-optical properties, terahertz time-domain spectroscopy, monolayer and bilayer molybdenum disulfide, nitrogen-doped carbon nanodots, hexagonal boron nitride

## Abstract

Terahertz (THz) radiation lies between the microwave and infrared (0.1–10 THz, photon energies ~0.4–40 meV), an energy scale matching the low-energy excitations and picosecond (ps) relaxation of free carriers; this makes THz magneto-optical (MO) spectroscopy a powerful, contactless probe of carrier dynamics in electronic materials. In this review, we summarize the progress in using THz time-domain spectroscopy (TDS) in the Faraday geometry to study the MO properties of low-dimensional and wide-bandgap materials. We first introduce the experimental principles and the theoretical framework, centered on the generalized MO Drude–Smith model that accounts for photon-induced electronic backscattering, and outline how the longitudinal conductivity and the left- and right-handed circularly polarized MO conductivities or conductances are extracted. We also describe how key electronic parameters, the carrier density, relaxation time, effective mass, static dielectric constant, and localization factor, are determined, with the effective mass obtained under a non-cyclotron resonance condition. We then review and compare studies on representative systems, including monolayer and bilayer MoS_2_, nitrogen-doped carbon nanodots (N-CNDs), nitrogen-doped diamond, and neutron-irradiated hexagonal boron nitride (hBN). Across these systems, common magnetic-field trends emerge: the carrier density and localization factor decrease, while the relaxation time, effective mass, and dielectric constant increase. For nitrogen-doped diamond, the magnetic-field dependences of the Faraday rotation angle and ellipticity reveal a pronounced THz MO response, highlighting its potential as a THz polarization-rotation material. Finally, we discuss the current challenges and future prospects, including broader spectral coverage, higher magnetic fields, spatially and time-resolved measurements, temperature and carrier-density control, material-specific modeling, and THz MO devices. This review highlights THz MO spectroscopy as a versatile tool for probing carrier dynamics in low-dimensional and wide-bandgap materials.

## 1. Introduction

The electronic and optoelectronic properties of materials are governed to a large extent by the dynamics of their mobile charge carriers [1]. Key parameters, including carrier density, momentum-relaxation time, mobility, effective mass, and carrier localization, determine the electrical conductivity and optical response and ultimately influence the performance of electronic, optoelectronic, and sensing devices [2,3]. Accurate determination of these parameters is particularly important, but often challenging, in both low-dimensional and wide-bandgap materials. In atomically thin two-dimensional (2D) crystals and zero-dimensional (0D) nanostructures, quantum confinement, reduced dielectric screening, and enhanced surface and interface effects can substantially modify carrier transport [4]. In wide-bandgap materials, low carrier densities, defect-mediated conduction, and high electrical resistivity can similarly complicate the determination of carrier properties [5]. Conventional electrical measurements require reliable Ohmic contacts, which are often difficult to fabricate on atomically thin layers, granular nanoparticle films, and chemically inert solids. Moreover, contact resistance, interface barriers, and processing-induced disorder may obscure the intrinsic material response. These limitations highlight the need for a contactless technique capable of probing free-carrier dynamics without patterned electrodes or electrical contacts [4].

**Figure 1:**
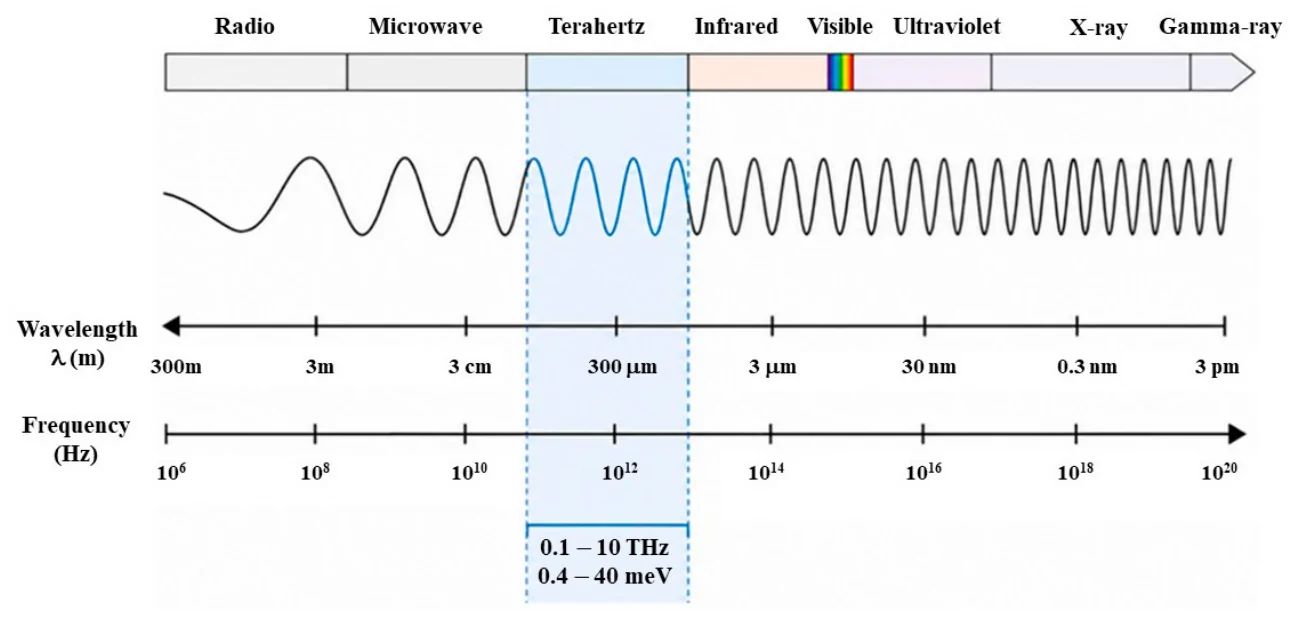
Position of the THz region within the electromagnetic spectrum. The conventional THz range of 0.1–10 THz corresponds to frequencies of 10^11^–10^13^ Hz, wavelengths of approximately 3 mm − 30 μm, and photon energies of approximately 0.4–40 meV.

Terahertz (THz) time-domain spectroscopy (TDS) is widely used to characterize the low-energy electrodynamic response of electronic materials [6,7]. The THz spectral range, conventionally defined as 0.1–10 THz, corresponds to photon energies of approximately 0.4–40 meV and electric-field oscillation periods on the picosecond timescale [7,8]. The position of this spectral window within the electromagnetic spectrum and its corresponding wavelength and photon-energy ranges are illustrated in Figure 1. These energy and timescales are well suited to probing intraband carrier motion, momentum relaxation, low-energy collective excitations, and disorder-induced carrier localization. Consequently, the THz electric field couples sensitively to the motion of mobile charge carriers. Because THz-TDS measures both the amplitude and phase of the transmitted electric field, the complex optical conductivities/conductances can be determined without applying a Kramers–Kronig transformation [9]. Analysis using the Drude, Drude–Smith, or related conductivity models can provide key carrier parameters, including carrier density, momentum-relaxation time, mobility, and the degree of carrier backscattering or localization [10]. THz-TDS has therefore become a powerful contactless technique for investigating carrier dynamics in metals, semiconductors, superconductors, nanostructures, and 2D materials [11].

THz-TDS can also be combined with an external magnetic field and low-temperature facilities to investigate the magneto-optical properties of electronic materials [10]. In the Faraday geometry, the magnetic field is parallel to the direction of THz propagation and normal to the sample surface. The Lorentz force induces transverse carrier motion characterized by the cyclotron frequency, *ω*_c_ = *qB*/m*, where *q* is the carrier charge, *B* is the magnetic flux density, and m* is the effective mass. The polarization-resolved THz fields can be transformed into left- and right-handed circularly polarized components [2]. Depending on the sample geometry and electrodynamic model, these components can be used to determine the circular conductivities (*σ*_±_ (*ω*))/conductances (*G*_±_ (*ω*)), or dielectric functions (*ϵ*_±_ (*ω*)). Drude-like responses can be analyzed using the conventional magneto-optical (MO) Drude model, whereas non-Drude spectra can be treated using the generalized MO Drude–Smith model [12]. Simultaneous analysis of the circular responses, or, equivalently, the longitudinal and transverse components, can identify the carrier type and cyclotron frequency and provide model-dependent estimates of the effective mass and magnetic-field-dependent transport parameters.

Despite these capabilities, THz MO investigations of low-dimensional and wide-bandgap materials remain comparatively scarce and are largely confined to material-specific studies. Direct comparison among these studies is not straightforward because the investigated systems differ substantially in dimensionality, sample configuration, transport regime, and electrodynamic treatment. The challenge becomes particularly significant when cyclotron resonance is not resolved within the accessible spectral window, because the effective mass and other carrier parameters must then be inferred indirectly by fitting the full complex response. A focused synthesis is therefore required to place the available results within a common analytical framework, distinguish directly observed spectral behavior from model-dependent quantities, and critically assess the reliability and generality of the extracted parameters. Such an assessment will also clarify the broader utility of THz MO spectroscopy for characterizing low-dimensional, doped, and irradiation-modified materials [13] and for informing the development of magnetically tunable THz polarization-control components [14,15].

This review examines the application of THz MO spectroscopy and the generalized MO Drude–Smith framework to representative low-dimensional and wide-bandgap materials, including monolayer and bilayer MoS_2_ [16,17], nitrogen-doped carbon nanodots (N-CNDs) [4], nitrogen-doped diamond [18], and neutron-irradiated hexagonal boron nitride (hBN) [5]. These systems provide complementary case studies spanning 2D semiconductors, assemblies of 0D nanostructures, and bulk wide-bandgap materials with different carrier types, disorder characteristics, and sample configurations. We present a consistent procedure for converting polarization-resolved THz measurements into circular conductivities, sheet conductances, or dielectric functions and critically compare the carrier parameters obtained for the different systems. Particular attention is given to non-resonant effective-mass determination, the interpretation of the photoinduced electronic localization parameter, and the extent to which the reported magnetic-field-dependent trends represent material behavior rather than consequences of parameter correlations or model assumptions.

The remainder of this review is organized as follows. Section 2 introduces the experimental principles of THz MO time-domain spectroscopy, summarizes the Drude, Drude–Smith, and generalized MO Drude–Smith models, and describes the extraction of the appropriate response functions for different sample configurations. Section 3 discusses applications to low-dimensional materials, whereas Section 4 focuses on bulk wide-bandgap systems. Section 5 presents a cross-material comparison and evaluates the principal experimental and modeling limitations. Finally, Section 6 summarizes the main conclusions and outlines directions for the further development and application of THz MO spectroscopy.

## 2. THz Magneto-Optical Spectroscopy: Principles and Analysis

This section describes the experimental and analytical principles of THz MO spectroscopy. We first describe polarization-resolved THz TDS in the Faraday geometry. We then explain how the measured THz fields are decomposed into left- and right-handed circularly polarized components and converted into the MO conductivities/conductances, or dielectric response for different sample geometries. Finally, the Drude, Drude–Smith, and MO Drude–Smith models used to analyze carrier transport, cyclotron motion, and carrier backscattering and localization factor.

### 2.1. THz-TDS in the Faraday Geometry

**Figure 2:**
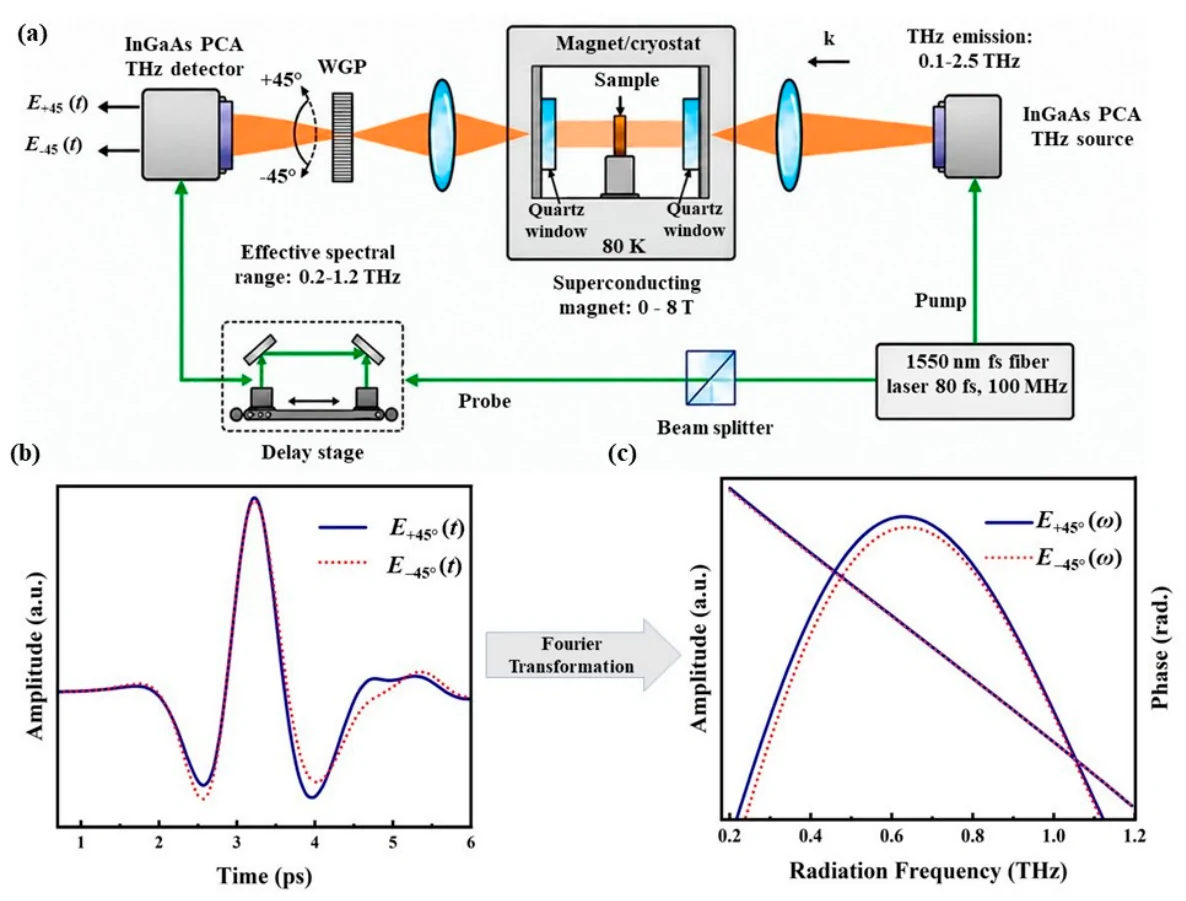
THz magneto-optical time-domain spectroscopy and signal processing. (**a**) Schematic of the THz-TDS system operated in Faraday geometry. THz pulses generated and detected by InGaAs photoconductive antennas pass through the sample inside an 80-K superconducting magnet (0–8 T). A wire-grid polarizer oriented at +45° and −45° enables measurement of the polarization-resolved electric fields. The quartz cryostat windows restrict the effective spectral range to 0.2–1.2 THz. (**b**) Representative time-domain waveforms, *E*_+45°_ (t) and *E*_−45°_ (t). (**c**) Corresponding frequency-domain amplitude and phase spectra obtained by Fourier transformation, *E*_+45°_ (ω) and *E*_−45°_ (ω).

The THz MO measurements reviewed here are carried out in the conventional Faraday geometry, in which both the incident THz beam and the magnetic field are directed perpendicular to the sample surface [10]. In this configuration, the linearly polarized THz field couples to the free carriers through the magnetic field, giving rise to a measurable MO response. Figure 2 shows a schematic of the THz-TDS system, and the measurement proceeds as follows.

(i) A femtosecond (fs) fiber laser beam (1550 nm wavelength, 80 fs pulse duration, and 100 MHz repetition rate) is split into two beams that serve as the pump and probe sources, respectively. (ii) The pump beam is focused onto an InGaAs photoconductive antenna (PCA) to generate a highly linearly polarized, pulsed THz radiation with a spectral range of about 0.1–2.5 THz. (iii) The THz beam is collimated and focused onto the sample, which is mounted on a sample holder in a cryostat fitted with quartz windows. Because the quartz windows absorb the high-frequency THz waves [12], the effective spectral range of the measurement is limited to approximately 0.2–1.2 THz. (iv) A superconducting magnet is used to tune the magnetic field B, applied parallel to the propagation direction of the THz beam and perpendicular to the sample surface, typically from 0 to 8 T. (v) After a variable time delay introduced by a delay stage, the probe beam is focused onto a second InGaAs PCA to detect, by electro-optical sampling, the THz field transmitted through the sample, which is recorded as a function of the delay time. (vi) The polarization of the transmitted beam is analyzed with a wire-grid polarizer (WGP), the angle of which is set to +45° and −45° with respect to the polarization of the linearly polarized incident beam; these two measurements provide the data required to extract the circularly polarized components.

To minimize the influence of THz absorption by moisture and dust, the key part of the system is sealed within a dry nitrogen-gas environment. The measurements reviewed here are conducted at a fixed liquid-nitrogen temperature of 80 K, with the magnetic field varied from 0 to 8 T.

### 2.2. Extraction of the Magneto-Optical Conductivities

In a THz MO measurement, the time-dependent THz electric field transmitted through the sample, *E*_±45°_(*t*), is first recorded at the +45° and −45° polarizer angles for each magnetic field [4]. For each sample, a reference is taken by measuring, at B = 0 T, the transmission through the same configuration without the active layer, for example, the bare substrate, or the two substrates of a sandwich structure. The use of a zero-field reference is justified because the sapphire substrates and the quartz cryostat windows possess a very small Verdet coefficient that depends only weakly on temperature, so that they do not themselves rotate the polarization of the THz beam in the presence of a magnetic field [19,20]. By Fourier transformation of these time-domain data, the corresponding complex electric fields *E*_±45°_ (*ω*) are obtained in the frequency domain, providing both the amplitude and the phase at each frequency [1].

According to the Fresnel theory, a linearly polarized field can be decomposed into left- and right-handed circularly polarized (LHCP and RHCP) components, which are obtained from the +45° and −45° fields of the sample or reference via [21],

(1) $$\left[\begin{array}{l} E_{+}^{\;q}\left(\omega \right)\\ E_{-}^{\;q}\left(\omega \right) \end{array}\right]=\frac{1}{2}\left(\begin{array}{cc} -1+i & 1+i\\ 1+i & -1+i \end{array}\right)\times \left[\begin{array}{l} E_{+45^{\circ }}^{q}\left(\omega \right)\\ E_{-45^{\circ }}^{q}\left(\omega \right) \end{array}\right]$$

where the superscript *q* denotes the reference or the sample, and the subscripts + and − denote the LHCP and RHCP components, respectively. The ratio of the corresponding sample and reference fields gives the LHCP and RHCP transmission coefficients *t*_±_ (*ω*). The relation between *t*_±_ (*ω*) and the MO conductivities (or conductance’s) is determined by the geometry of the sample. Three cases are relevant to the materials reviewed here: a thin film on a substrate [16,17], a conducting layer sandwiched between two substrates [4], and a bulk sample [5,18].

#### 2.2.1. Thin Film on a Substrate

An atomically thin conducting film deposited on a dielectric substrate can be treated as a two-dimensional conducting sheet because its thickness is negligible compared with the THz wavelength. Within the Tinkham approximation [22], the complex transmission normalized to that of the bare substrate is related directly to the sheet conductivity. In the monolayer MoS_2_ study, only the longitudinal transmitted field was measured; hence,

(2) $$t_{\mathrm{xx}}=\frac{1+n_{s}}{1+n_{s}+Z_{0}\sigma_{\mathrm{xx}}(\omega )}$$

For bilayer MoS_2_, the polarization-resolved transmitted fields were decomposed into LHCP and RHCP components, and the corresponding circular transmission coefficients were expressed as

(3) $$t_{\pm }(\omega )=\frac{1+n_{s}}{1+n_{s}+Z_{0}\sigma_{\pm }(\omega )}$$

Here, *n_s_* = 3.07 is the refractive index of the substrate [23], *Z*_0_ = 377 Ω is the impedance of free space [24], *σ_xx_* (*ω*) is the complex longitudinal sheet conductivity, and *σ*_±_ (*ω*) denotes the complex circular sheet conductivities. These relations can be inverted directly to obtain the corresponding conductivity from the measured complex transmission. Thus, the same thin-film electrodynamic approximation is used for both MoS_2_ systems, but the response is expressed in the longitudinal basis for monolayer MoS_2_ and in the circular basis for bilayer MoS_2_ [16,17].

#### 2.2.2. Conducting Layer Between Two Substrates

For the air–substrate–conducting-layer–substrate–air configuration considered for the N-CND sample, the two sapphire substrates were taken to be identical. The circular transmission coefficient was defined as the ratio of the THz field transmitted through the sapphire/N-CND/sapphire assembly to that transmitted through two sapphire substrates without the N-CND layer. It is approximated by [4],

(4) $$t_{\pm }(\omega )=\frac{2n}{2n+Z_{0}G_{\pm }(\omega )}$$

*G*_±_ (*ω*) = *G*_1±_ (*ω*) + *iG*_2±_ (*ω*) denotes the complex LHCP or RHCP magneto-optical conductance of the N-CND layer. This configuration was adopted for the N-CND layer enclosed between two sapphire substrates. A similar sandwich structure was previously used for THz-TDS measurements of fluorescent carbon quantum dots in the absence of a magnetic field [25].

#### 2.2.3. Bulk Sample

For a bulk sample with a thickness *d* larger than the wavelength of the THz radiation, the relation between the transmission coefficient and the complex refractive index refractive *n*_±_(*ω*) = *n*_±1_(*ω*) + *in*_±2_(*ω*) [26],

(5) $$t_{\pm }(\omega )=\frac{E_{\pm }^{\mathrm{sam}+\mathrm{sub}}(\omega )}{E_{\pm }^{\mathrm{sub}}(\omega )}=\frac{4n_{\pm }(\omega )}{{\left[1+n_{\pm }(\omega )\right]}^{2}}e^{\mathrm{i\omega d}(n_{\pm }(\omega )-1)/c}$$

where *d* is the sample thickness, *c* is the speed of light in vacuum. In the polar form $t_{\pm }\;\left(\omega \right)=A_{\pm \;}(\omega )e^{-i\phi_{\pm \;}(\omega )}\;$, where *A*_±_ (*ω*), and *ϕ*_±_ (*ω*) are, respectively, the amplitude and the phase of the complex LHCP or RHCP transmission coefficient,

(6) $$A_{\pm }(\omega )\approx \frac{4n_{\pm 1}(\omega )}{{\left[n_{\pm 1}(\omega )+1\right]}^{2}}e^{-\mathrm{\omega d}n_{\pm 2}(\omega )/c},\phi_{\pm }(\omega )\approx \frac{\mathrm{\omega d}[n_{\pm 1}(\omega )-1]}{c},$$

For the case of weak optical absorption, i.e., *n*_±1_ (*ω*)≫*n*_±2_ (*ω*) [26], Equation (5) reduces to

(7) $$n_{\pm 1}(\omega )≃1+\frac{c\varphi_{\pm }(\omega )}{\mathrm{\omega d}},n_{\pm 2}(\omega )≃-\frac{c}{\mathrm{\omega d}}\ln\left[A_{\pm }(\omega )\frac{{\left[n_{\pm 1}(\omega )+1\right]}^{2}}{4n_{\pm 1}(\omega )}\right]$$

The complex dynamic dielectric function *ε*_±_ (*ω*) and the MO conductivity *σ*_±_ (*ω*) are then related by [10],

*ε*_±_(*ω*) = *n*_±_^2^(*ω*) = *ε_b_* + *iσ*_±_(*ω*)/(*ωε*_0_), *σ*_±_(*ω*) = *σ*_±1_(*ω*) + *iσ*_±2_(*ω*) (8)

*ε_b_* is the static (background) dielectric constant and *ε*_0_ is the permittivity of vacuum. In this case, the static dielectric constant *ε_b_* is obtained in addition to the MO conductivity. This route is used for the bulk nitrogen-doped diamond and neutron-irradiated hBN samples [5,18].

#### 2.2.4. Faraday Rotation Angle and Ellipticity

In the presence of a magnetic field, differences between the LHCP and RHCP components can rotate the polarization axis of the transmitted THz field and produce elliptical polarization. Using the convention adopted here, the Faraday rotation angle and ellipticity are calculated as [27]:

(9) $$\theta (\omega )=\left\{\arg\left[E_{+}^{f}(\omega )\right]-\arg\left[E_{-}^{f}\left(\omega \right)\right]\right\}/2$$

(10) $$\eta (\omega )=\frac{\left|E_{+}^{f}(\omega )\right|-\left|E_{-}^{f}(\omega )\right|}{\left|E_{+}^{f}(\omega )\right|+\left|E_{-}^{f}(\omega )\right|}$$

Here $E_{\pm }^{f}(\omega )$ denotes the normalized complex circular transmission coefficient, with $\left|E_{\pm }^{f\;}(\omega )\right|=$ $\left|E_{\pm }^{\mathrm{fs}}\;(\omega )\right|/\left|E_{\pm }^{s}\;(\omega )\right|$, and $\arg\left[\;E_{\pm \;}^{f}\left(\omega \right)\right]=\arg\left[E_{\pm }^{\mathrm{fs}}\;(\omega )\right]-\arg\left[E_{\pm }^{s}\;\left(\omega \right)\right],$ where the superscripts *fs* and *s* denote the sample structure and the corresponding substrate reference, respectively. The phase difference between the two circular components determines the Faraday rotation angle, whereas their normalized amplitude difference determines the ellipticity [28]. This methodology is applied to the nitrogen-doped diamond results discussed in Section 4.1.2.

### 2.3. Theoretical Framework

A quantitative interpretation of the measured THz response requires transport models that connect the complex spectra with the underlying carrier dynamics. The conventional Drude model provides the reference description of freely conducting carriers [29], whereas the Drude–Smith model accounts phenomenologically for preferential backscattering and restricted carrier motion [12]. Depending on the measured response and sample geometry, fitting yields the carrier density or density-related coefficient, relaxation time, effective mass and localization factor and, for bulk samples, the static dielectric constant. The optical and magneto-optical forms of these models used in the reviewed studies are introduced below.

#### 2.3.1. Drude and Drude–Smith Models

The Drude model describes the motion of free electrons as a classical free-electron gas and is widely used to characterize the carrier transport and optical conductivity of metals and semiconductors [29]. In the absence of an external magnetic field, the frequency dependence of the complex conductivity is given by

(11) $$\sigma (\omega )=\frac{\sigma_{0}}{1-\mathrm{i\omega \tau}},$$

where *ω* = 2*πf* is the angular frequency of THz radiation, τ is the carrier relaxation time, and *σ*_0_ = *ne*^2^*τ*/m^*^ is the DC conductivity. Here, *n* represents the concentration of free carriers, and m* denotes the effective mass of the electrons. The characteristics of this model are as follows: the real part *σ*_1_ (*ω*) = *σ*_0_/(1 + *ω*^2^*τ*^2^) reaches its maximum at zero frequency and decreases with increasing frequency. On the other hand, its imaginary part *σ*_2_ (*ω*) = *ωτσ*_0_/(1 + *ω*^2^*τ*^2^), remains positive at all frequencies.

However, the conductivities of some metals and semiconductors cannot be described by the conventional Drude model. The model was therefore extended, on the basis of the pulse-response method and the Poisson statistics, to the Drude–Smith model [12], which accounts for the optically induced localization and backscattering of free carriers. In this extension, the collisions experienced by an electron are assumed to be randomly distributed in time, with an average interval *τ* between collisions, and a localization factor α_n_ is introduced into the Drude model [12],

(12) $$\sigma (\omega )=\frac{\sigma_{0}}{1-\mathrm{i\omega \tau}}\left[1+\sum_{n=1}^{\infty }\frac{\alpha_{n}}{{\left(1-\mathrm{i\omega \tau}\right)}^{n}}\right]$$

where *α*_n_ = [−1, 0] represents the fraction of the electron’s velocity retained after its *n*th collision relative to its initial velocity. When α_n_ = 0, the Drude–Smith model reduces to the Drude model; when α_n_ = −1, the electrons tend toward complete localization, and both the real and imaginary parts of the conductivity vanish as ω → 0. The introduction of α_n_ allows the imaginary part of the conductivity to take negative values. In practice, retaining only the first backscattering event (*n* = 1) provides a good fit to the experimental data, in which case the Drude–Smith model is written as [12]

(13) $$\sigma (\omega )=\frac{\sigma_{0}}{1-\mathrm{i\omega \tau}}\left[1+\frac{\alpha }{\left(1-\mathrm{i\omega \tau}\right)}\right]$$

#### 2.3.2. Magneto-Optical Drude and Drude–Smith Models

In a THz-TDS measurement with the magnetic field configured in the Faraday geometry, the THz polarization measurements at +45° and −45° allow the left-handed and right-handed circularly polarized conductivities of the sample to be determined experimentally, and a corresponding theoretical model is required for their analysis. By introducing the cyclotron frequency *ω*_c_ = *eB*/m*, where *B* is the magnetic field strength, into the Drude and Drude–Smith models, the corresponding magneto-optical conductivity models are obtained. The magneto-optical Drude model is given by [12]

(14) $$\begin{array}{l} \sigma_{\mathrm{xx}}(\omega )=\frac{\sigma_{0}(1-\mathrm{i\omega \tau})}{{\left(1-\mathrm{i\omega \tau}\right)}^{2}+{\left(\omega_{c}\tau \right)}^{2}}\\ \sigma_{\mathrm{xy}}(\omega )=\frac{\left(\sigma_{0}\omega_{c}\tau \right)}{{\left(1-\mathrm{i\omega \tau}\right)}^{2}+{\left(\omega_{c}\tau \right)}^{2}} \end{array}$$

where *σ*_xx_ and *σ*_xy_ are the longitudinal and transverse magneto-optical conductivities, respectively. The relationships among the longitudinal, transverse, and circularly polarized conductivities are [30]

*σ*_±_(*ω*) = *σ_xx_*(*ω*) ± *iσ_xy_*(*ω*) (15)

From Equation (15), the left-handed and right-handed circularly polarized conductivities for the magneto-optical Drude model are expressed as [30]

(16) $$\sigma_{\pm }(\omega )=\frac{\sigma_{0}}{1-i(\omega \mp \omega_{c})\tau }$$

The Drude–Smith model, which includes the backscattering and localization of free carriers, was extended to its magneto-optical form by our group in 2016 [10]. Considering only the first collision, the magneto-optical Drude–Smith model gives the longitudinal and transverse conductivities

(17) $$\begin{array}{l} \sigma_{\mathrm{xx}}(\omega )=\frac{\sigma_{0}}{{\left(1-\mathrm{i\omega \tau}\right)}^{2}+{\left(\omega_{c}\tau \right)}^{2}}\left[1-\mathrm{i\omega \tau}+\alpha -\frac{2\alpha {\left(\omega_{c}\tau \right)}^{2}}{{\left(1-\mathrm{i\omega \tau}\right)}^{2}+{\left(\omega_{c}\tau \right)}^{2}}\right]\\ \sigma_{\mathrm{xy}}(\omega )=\frac{\sigma_{0}\omega_{c}\tau }{{\left(1-\mathrm{i\omega \tau}\right)}^{2}+{\left(\omega_{c}\tau \right)}^{2}}\left[1+\frac{2\alpha (1-\mathrm{i\omega \tau})}{{\left(1-\mathrm{i\omega \tau}\right)}^{2}+{\left(\omega_{c}\tau \right)}^{2}}\right] \end{array}$$

Combining the magneto-optical Drude–Smith model with Equation (15), the left-handed and right-handed circularly polarized conductivities (or conductances) are determined as [10]

(18) $$\sigma_{\pm }(\omega )=\frac{\sigma_{0}}{1-i(\omega \mp \omega_{c})\tau }\left[1+\frac{\alpha }{1-i(\omega \mp \omega_{c})\tau }\right]$$

Here, *α* is the first-order electronic localization factor. By fitting the measured *σ*_±_ (*ω*), or *G*_±_ (*ω*), with Equation (18), the carrier density *n* (or the coefficient γ = *gn* when the geometry factor *g* is unknown), the relaxation time *τ*, the localization factor *α*, and the cyclotron frequency *ω*_c_ are obtained; the effective mass follows from *ω*_c_ = *eB*/m*, the mobility from *μ* = *eτ*/m*, and, for the bulk samples, the static dielectric constant *ε*_b_ is obtained in addition.

## 3. Low-Dimensional Semiconductors

Reduced dimensionality can strongly modify the low-energy carrier response through changes in electronic structure, interlayer coupling, interfaces, finite size, and structural disorder [31]. This section considers two complementary classes of low-dimensional semiconductors: atomically thin MoS_2_ and nitrogen-doped carbon nanodots (N-CNDs). Monolayer and 2H-stacked bilayer MoS_2_ provide a direct comparison of layer-dependent transport in crystalline two-dimensional semiconductors [16,17], whereas N-CNDs extend the discussion to a granular layer composed of quasi-zero-dimensional carbon nanostructures [4].

The measured electrodynamic quantities differ according to the sample geometry and experimental configuration. The monolayer MoS_2_ study examined the magnetic-field-dependent longitudinal conductivity *σ*_xx_ (*ω*), whereas the bilayer study resolved the left- and right-handed circular conductivities, *σ*_±_ (*ω*). For the N-CND layer, the corresponding circular sheet conductances, *G*_±_ (*ω*), were determined. The following subsections discuss these material-specific THz magneto-optical responses and their magnetic-field dependences using the analytical framework established in Section 2.

### 3.1. Molybdenum Disulfide

Molybdenum disulfide (MoS_2_) is one of the most extensively investigated transition-metal dichalcogenides (TMDs) [32], owing to its layer-dependent electronic structure and the pronounced changes in its optical and transport properties with thickness [33]. In the monolayer limit, MoS_2_ is a direct band gap semiconductor with band extrema at the *K* and *K*′ valleys [34,35]. However, increasing the number of layers modifies the band structure through interlayer coupling and results in an indirect-gap electronic structure in the bilayer [36,37]. In the latter, the conduction-band minimum is located near the *Q* point between *Γ* and *K* [38]. These changes can alter the distribution, effective mass, and scattering dynamics of low-energy carriers, making MoS_2_ a suitable system for examining the relationship between layer-dependent electronic structure and magneto transport.

THz magneto-optical investigations of monolayer and bilayer MoS_2_ provide complementary information on how these structural and electronic changes influence intraband carrier dynamics under an applied magnetic field [16,17]. The following subsections consider the two systems separately, relating their structural and optical characterization to the measured THz response and the field dependence of the extracted carrier parameters.

#### 3.1.1. Monolayer MoS_2_

**Figure 3:**
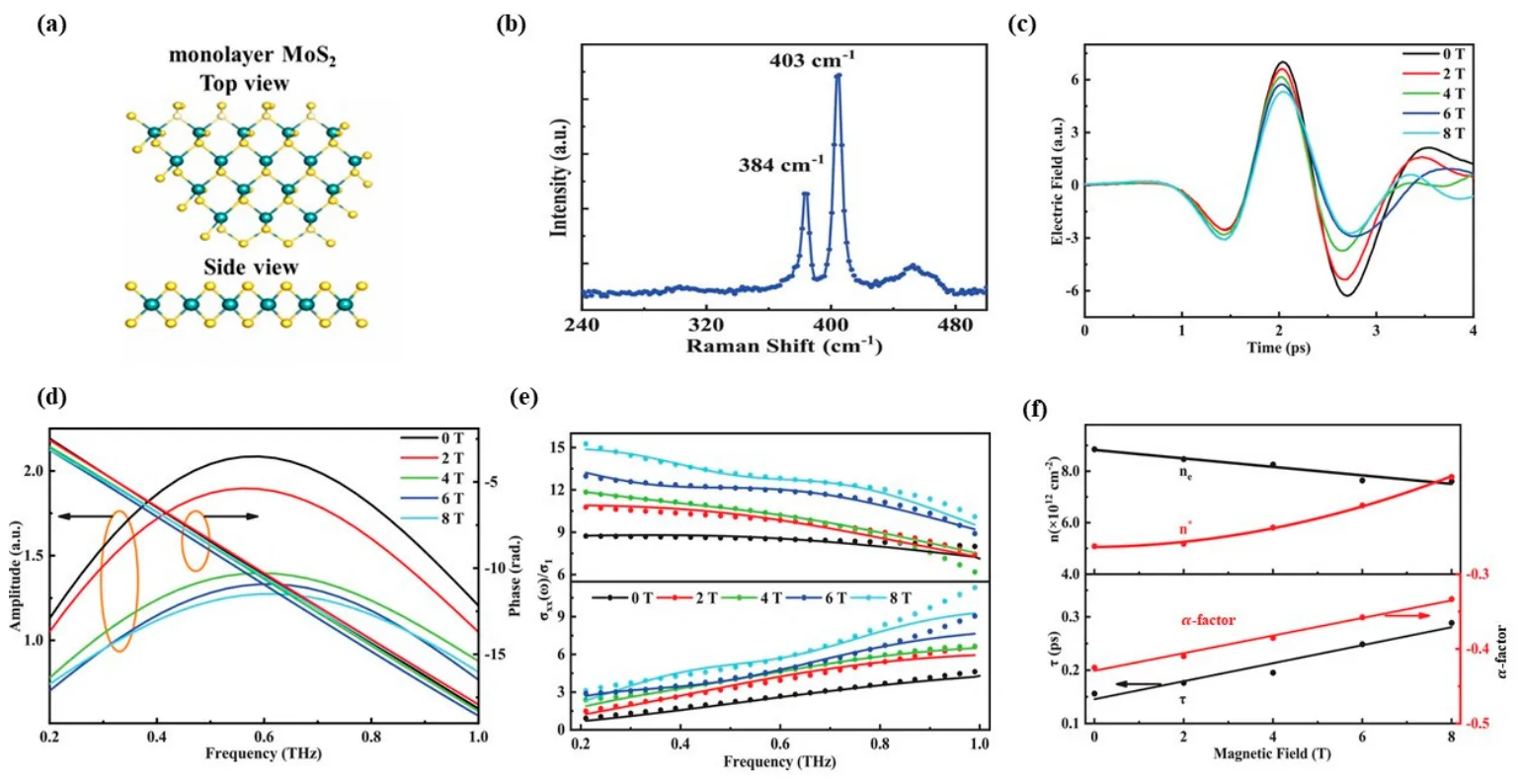
THz magneto-optical response of monolayer MoS_2_. (**a**) Top and side views of the monolayer lattice. (**b**) Raman spectrum supporting the monolayer character of the MoS_2_ film. (**c**) Transmitted THz time-domain waveforms and (**d**) corresponding frequency-domain amplitude and phase spectra measured at 80 K under magnetic fields of 0–8 T. (**e**) Real and imaginary components of the longitudinal magneto-optical conductivity; symbols represent experimental results and curves represent generalized magneto-optical Drude–Smith fits. (**f**) Extracted electron density *n*_e_, static effective electron density n*, relaxation time *τ*, and electronic localization factor α as functions of magnetic field [16].

The THz magneto-optical response of CVD-grown monolayer MoS_2_ was investigated after transferring the film onto a SiO_2_/Si substrate [16,39]. The monolayer lattice structure is illustrated in Figure 3a. Optical microscopy indicated a continuous film with a relatively uniform surface, while Raman spectroscopy supported its monolayer character as shown in Figure 3b [39,40], while photoluminescence exhibited an emission peak near 1.86 eV, consistent with its direct-gap optical response [41,42].

The magneto-optical measurements were performed at 80 K using the Faraday geometry described in Section 2.1, over an effective spectral range of approximately 0.2–1.0 THz and under magnetic fields from 0 to 8 T [16]. The transmitted THz time-domain waveforms and corresponding frequency-domain amplitude and phase spectra are shown in Figure 3c,d, respectively. The transmitted THz amplitude decreased systematically with increasing magnetic field, whereas the transmission through the bare SiO_2_/Si substrate was almost field independent. The observed field dependence could therefore be assigned predominantly to the MoS_2_ layer. As the detection configuration selected the transmitted field component parallel to the incident polarization, the experiment measured the longitudinal response. The normalized transmission through the MoS_2_-SiO_2_/Si structure was converted into the complex longitudinal conductivity, *σ*_xx_ (ω), using the longitudinal form of the thin-film Tinkham relation introduced in Section 2.2.1. Since the THz photon energies were substantially below the approximately 1.8 eV band gap of monolayer MoS_2_, the measured conductivity was attributed to intraband motion of existing free carriers rather than interband excitation [2].

As shown in Figure 3e, the real and imaginary components of the longitudinal conductivity, *σ*_1_ (*ω*) and *σ*_2_ (*ω*), increased in magnitude with increasing magnetic field. With increasing frequency, *σ*_1_ (*ω*) generally decreased, whereas *σ*_2_ (*ω*) increased. The real component additionally exhibited a weak non-monotonic frequency dependence that became more pronounced at high magnetic fields. This spectral feature was not identified as a cyclotron resonance [16].

The conventional magneto-optical Drude model [2] could not reproduce the complete frequency dependence of the measured conductivity, particularly the non-monotonic behavior of its real component. In contrast, the generalized magneto-optical Drude–Smith model [10] provided a consistent description of both *σ*_1_ (*ω*) and *σ*_2_ (*ω*) over the investigated magnetic-field range. The negative electronic localization factor α indicates the presence of preferential carrier backscattering or restricted carrier motion. Within this model, *α* = 0 corresponds to the conventional Drude limit, whereas values approaching −1 represent increasingly strong carrier localization.

For the fitting analysis, the electron effective mass was taken as m* = 0.39 *m*_0_ [43], where *m*_0_ is the free-electron mass. The electron density *n*_e_, relaxation time *τ*, and localization parameter *c* were determined from the field-dependent conductivity spectra [16]. The fitted parameters are summarized in Figure 3f. At zero magnetic field, the extracted electron density was approximately 8.83 × 10^12^ cm^−2^. With increasing magnetic field, *n*_e_ gradually decreased, reaching approximately 7.51 × 10^12^ cm^−2^ at 8 T. In contrast, the relaxation time exhibited the opposite trend. It increased from approximately 0.145 ps at 0 T to 0.279 ps at 8 T. The fitted decrease in *n*_e_ should be interpreted together with the simultaneous changes in *τ* and *c*, because these parameters collectively determine the measured conductivity.

The electronic localization parameter changed from approximately −0.429 at zero field to −0.335 at 8 T. Thus, α became less negative and its magnitude decreased with increasing magnetic field, although the response remained outside the conventional Drude limit of α = 0. Within the Drude–Smith description, the reduction in ∣*α*∣ indicates weaker preferential backscattering or less restricted carrier motion. The authors attributed the simultaneous increase in *τ* and decrease in ∣*α*∣ to magnetic-field-induced orbital motion, which can reduce the probability of repeated carrier backscattering [2].

The study further distinguished the fitted electron density *n*_e_ from the static effective electron density n*, which incorporates the effects of magnetic field, relaxation time, and localization on the zero-frequency conductivity. Although *n*_e_ decreased with field, n* increased from approximately 5.05 × 10^12^ cm^−2^ at zero field to 7.8 × 10^12^ cm^−2^ at 8 T. At sufficiently high fields, n* therefore exceeded *n*_e_. This behavior does not imply an increase in the actual carrier population; rather, it reflects the combined field dependence of *n*_e_, *τ* and *α* in determining the effective static transport response [16].

Microstructural effects provide complementary context for interpreting non-Drude transport. Han et al. investigated the zero-field THz conductivity of epitaxial monolayer MoS_2_ bicrystals containing mirror twin boundary networks [44]. Drude–Smith analysis, together with electrical transport measurements, supported conduction through interconnected quasi-metallic boundary channels with partial carrier localization. These findings illustrate that a negative Drude–Smith parameter does not uniquely identify the microscopic scattering mechanism. Accordingly, the reduction in |α| in Ref. [16] is consistent with less restricted carrier motion within the model, but its microscopic origin requires additional evidence. The different sample structures and absence of an applied magnetic field in Ref. [44] limit direct comparison with the magneto-optical results.

Despite the pronounced field dependence of the complex conductivity, no distinct cyclotron-resonance peak was observed within the measured spectral range. The study attributed its absence to the relatively large effective electron mass, limited carrier mobility, insufficient *ω_c_ τ*, and weakening of the resonance by carrier backscattering. Nevertheless, fitting the non-resonant longitudinal conductivity provided quantitative information on the field-dependent carrier density, relaxation time, and localization factor. The monolayer results therefore establish a non-Drude magneto-optical response characterized by an increasing relaxation time and decreasing ∣α∣ with magnetic field. The following subsection considers bilayer MoS_2_ using polarization-resolved measurements, from which the right- and left-handed circular conductivities and effective electron mass were additionally determined [17].

#### 3.1.2. Bilayer MoS_2_

**Figure 4:**
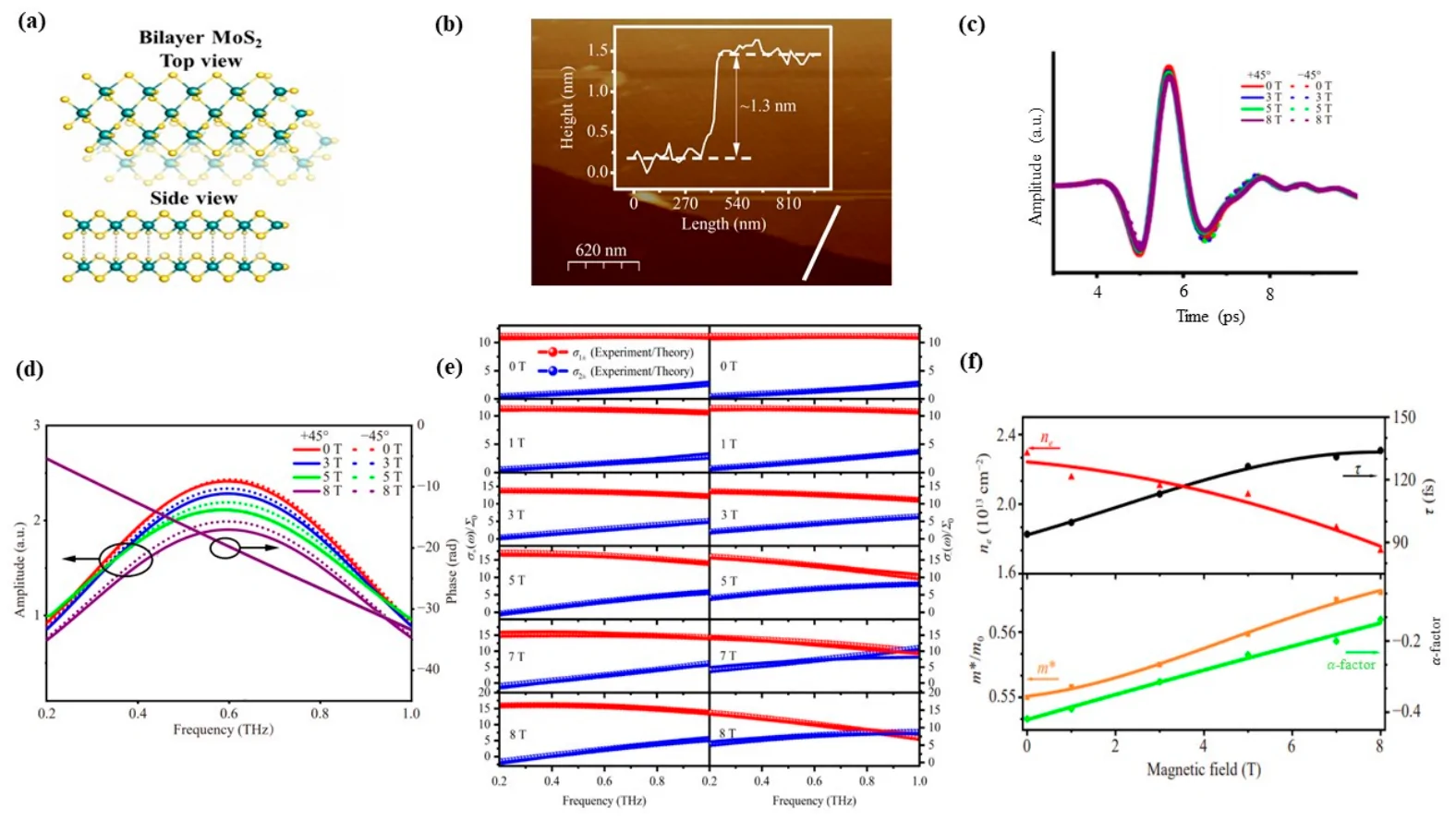
THz magneto-optical response of 2H-stacked bilayer MoS_2_. (**a**) Top and side views of the bilayer lattice. (**b**) AFM image and corresponding height profile indicating a film thickness of approximately 1.3 nm [45]. (**c**) Transmitted THz time-domain waveforms and (**d**) corresponding frequency-domain amplitude and phase spectra measured at analyzer angles of +45° and −45° under selected magnetic fields. (**e**) Real and imaginary components of the circular magneto-optical conductivities measured at 80 K under magnetic fields of 0–8 T; symbols represent experimental results and curves represent generalized magneto-optical Drude–Smith fits. (**f**) Extracted electron density *n*_e_, relaxation time τ, effective electron mass m*, and electronic localization factor *α* as functions of magnetic field. Panel (**b**) adapted with permission from Ref. [45]. Copyright © 2023 Higher Education Press. Panels (**c**–**f**) adapted with permission from Ref. [17]. Copyright © 2024 Higher Education Press.

The THz magneto-optical properties of n-type 2H-stacked bilayer MoS_2_ on sapphire were investigated using polarization-resolved THz-TDS [17]. The bilayer lattice structure is illustrated in Figure 4a. AFM measurements indicated a film thickness of approximately 1.3 nm, consistent with bilayer MoS_2_ as illustrated in Figure 4b. Complementary Raman and photoluminescence measurements supported the bilayer assignment, while Hall measurements established the n-type character of the sample [17,45,46].

Interlayer coupling substantially modifies the electronic structure of 2H-stacked bilayer MoS_2_. In contrast to monolayer MoS_2_, the conduction-band minimum occurs near the Q point between Γ and K. The energy difference between the Q- and K-derived conduction states was reported to be approximately 197 meV [38]. Because the THz photon energy within the experimental window is only a few millielectronvolts, the applied THz field cannot induce transitions between these valleys or generate electron–hole pairs. The measured response was therefore attributed predominantly to intraband dynamics of electrons occupying the Q valleys near the Fermi level [17].

The measurements were performed at 80 K using the polarization-resolved Faraday geometry described in Section 2.1, over an effective frequency range of approximately 0.2–1.0 THz and under magnetic fields from 0 to 8 T [17]. The transmitted THz time-domain waveforms and corresponding frequency-domain amplitude and phase spectra are shown in Figure 4c,d, respectively. Differences between the spectra measured at analyzer angles of +45° and −45° at finite magnetic fields indicated a field-induced modification of the transmitted polarization. Following Fourier transformation, the complex electric-field spectra measured at these analyzer angles were combined to obtain the left- and right-handed circular components using the procedure described in Section 2. The corresponding substrate-normalized circular transmission coefficients were then converted into the complex magneto-optical conductivities, *σ*_+_ (ω) and *σ*_−_ (ω), using the thin-film Tinkham relation introduced in Section 2.2.1.

The real and imaginary components of the circular conductivities are shown in Figure 4e. The difference between the two circular responses became more pronounced with increasing magnetic field. For the left-handed conductivity, *σ*_1−_ (*ω*) decreased markedly with frequency, whereas *σ*_2−_ (*ω*) increased. In contrast, the real and imaginary components of the right-handed conductivity, *σ*_+_ (*ω*), exhibited weaker frequency dependence over the measured spectral range. These spectra were fitted simultaneously using the generalized magneto-optical Drude–Smith model described in Section 2.3.2 [10], yielding the electron density *n*_e_, relaxation time τ, effective mass m*, and localization factor *α*, whose magnetic-field dependences are summarized in Figure 4f [17]. Unlike the monolayer study, in which m* was fixed during fitting of the longitudinal conductivity [16], the finite-field circular responses provided additional constraints for extracting the bilayer effective mass.

The extracted electron density decreased from approximately 2.3 × 10^13^ cm^−2^ at low field to about 1.7 × 10^13^ cm^−2^ at 8 T. This decrease was attributed to electronic localization under a strong magnetic field and charged-impurity scattering [47]. In contrast, the relaxation time increased from approximately 94 fs near zero field to slightly below 140 fs at 8 T. This increase was attributed to the decreasing cyclotron radius, which reduces the probability of effective carrier scattering from phonons and impurities as the magnetic field increases [47].

Near zero field, the electronic localization factor was approximately −0.418, indicating a pronounced deviation from the conventional Drude limit. With increasing magnetic field, *α* became progressively less negative and ∣*α*∣ decreased. Within the Drude–Smith description, this trend represents weaker preferential backscattering or less restricted carrier motion. This behavior was related to the increase in *τ*, because a longer interval between collision events reduces the persistence of the velocity change following a backscattering event.

The fitted zero-field values of *τ* ≈ 94 fs, m* ≈ 0.55 *m*_0_, and *α* ≈ −0.418 yielded an optically inferred longitudinal mobility of approximately 174 cm^2^V^−1^s^−1^ at 80 K. This value includes the reduction in the static mobility produced by the negative localization factor. The effective electron mass increased slightly with magnetic field, from approximately 0.55 *m*_0_ at low field to about 0.566 *m*_0_ at high field. This weak increase was attributed to magnetic confinement of the electron motion [48].

No distinct cyclotron-resonance feature was resolved in either circular conductivity. Its absence was attributed to the relatively large Q-valley effective mass and the short relaxation time, which remained below 0.14 ps even at 8 T [17]. Consequently, the condition *ωτ* ≈ *ω_c_ τ* > 1 required to observe cyclotron resonance was not satisfied within the measured frequency range. The study indicated that magnetic fields above 8 T and/or measurements extending beyond 1 THz would be required to resolve the resonance.

Despite the absence of cyclotron resonance, simultaneous fitting of the field-dependent circular conductivities yielded an effective electron mass of approximately 0.566 *m*_0_, close to the value obtained from density-functional calculations for electrons near the Q point. This was reported as the first experimental determination of the Q-valley effective mass in 2H-stacked bilayer MoS_2_ under a non-resonant condition. These results demonstrate the capability of polarization-resolved THz magneto-optical spectroscopy to determine carrier density, relaxation time, localization factor, and effective mass without a spectrally resolved cyclotron resonance.

For comparison, resolved cyclotron resonance in another two-dimensional carrier system illustrates the additional constraints provided by polarization-resolved THz magneto-optical spectroscopy. Poumirol et al. investigated electrostatically gated continuous graphene at 250 K under perpendicular magnetic fields up to 7 T [14]. THz transmission and Faraday-rotation measurements, combined with magneto-optical Kramers–Kronig analysis, yielded circularly polarized transmission spectra exhibiting a cyclotron resonance described by a semiclassical Drude model. At fixed carrier density, the resonance frequency increased linearly with magnetic field, while its dependence on carrier density was consistent with the density-dependent cyclotron mass of graphene. Switching between electron and hole doping reversed the Faraday rotation and magnetic circular dichroism at fixed magnetic field, demonstrating sensitivity to carrier polarity. This study provides a useful methodological benchmark for the information obtainable when cyclotron resonance is directly resolved. In contrast, no distinct cyclotron resonance was observed in the monolayer or bilayer MoS_2_ measurements [16,17], within the investigated spectral range, and the carrier parameters were therefore inferred by fitting the complex THz response using the generalized magneto-optical Drude–Smith model.

### 3.2. Nitrogen-Doped Carbon Nanodots

Carbon nanodots (CNDs) are surface-passivated zero-dimensional carbon nanostructures whose electronic and optical properties depend strongly on the size and geometry of the conjugated carbon domains and the chemical groups attached to their surfaces or edges [49]. Nitrogen doping is particularly relevant because it can produce n-type CNDs and modify their photoluminescence and THz response [50]. When deposited as a dried layer, N-CNDs form a granular and porous structure rather than a continuous crystalline film. Their measured conductance therefore contains contributions from both the individual nanodots and the overall morphology of the deposited layer. These characteristics make N-CNDs an interesting system for investigating electronic backscattering and localization in a quasi-zero-dimensional material [25].

**Figure 5:**
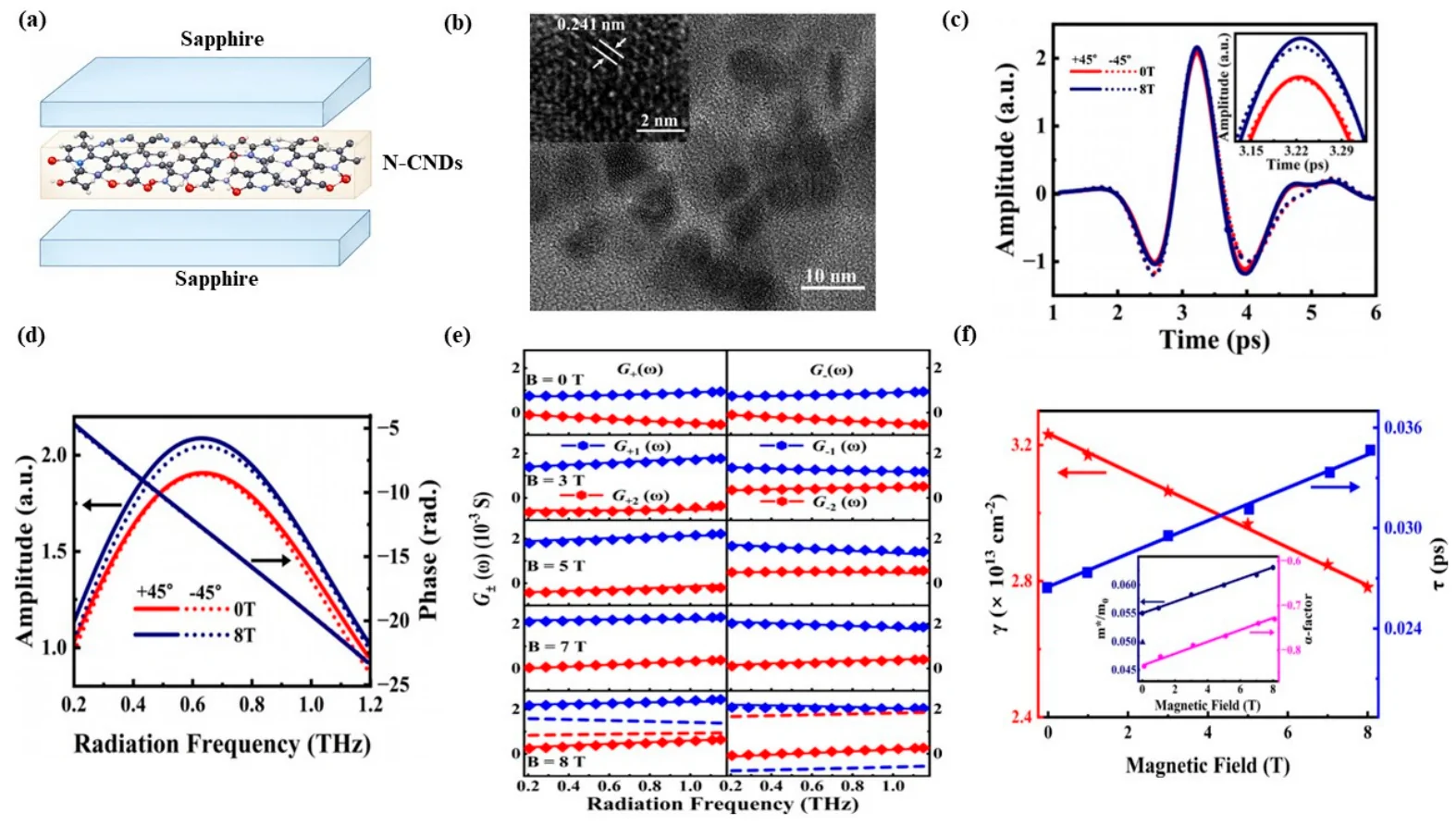
THz magneto-optical response of N-CNDs. (**a**) Schematic of the N-CND layer sandwiched between two sapphire substrates. (**b**) TEM image of the N-CNDs; the high-resolution inset shows lattice fringes with a spacing of approximately 0.241 nm. (**c**) Transmitted THz time-domain waveforms and (**d**) corresponding frequency-domain amplitude and phase spectra measured at 80 K at analyzer angles of +45° and −45° under magnetic fields of 0 and 8 T. The inset in (**c**) highlights the waveform maxima. (**e**) Real (blue) and imaginary (red) components of the circular sheet conductances, *G*_+_ (ω) and *G*_−_ (ω), under selected magnetic fields between 0 and 8 T; symbols represent experimental results and curves represent magneto-optical Drude–Smith fits. (**f**) Magnetic-field dependence of the density-related coefficient *γ* and relaxation time τ; the inset shows the normalized effective mass m*/*m*_0_ and localization factor α. Panels (**b**–**f**) reprinted with permission from Ref. [4]. Copyright © 2025 by the American Physical Society.

The N-CNDs were prepared by a hydrothermal method using citric acid as the carbon source, urea as the nitrogen source, and dimethyl sulfoxide as a passivation agent [25]. TEM measurements indicated an average nanodot diameter of approximately 5.5 nm, with lattice fringes suggesting local crystalline ordering as shown in Figure 5b. Complementary FTIR and photoluminescence measurements indicated surface functionalization and excitation-dependent emission [51,52,53,54]. For the THz-TDS measurements, an ethanol dispersion of the N-CNDs was deposited onto an approximately 0.5-mm-thick sapphire substrate and dried to form a particulate layer. A second sapphire substrate of similar thickness was placed over the layer, producing the sapphire/N-CND/sapphire structure illustrated in Figure 5a [4].

The role of chemical modification and structural disorder in carbon-dot transport was examined by Song et al. through zero-field THz-TDS measurements of graphene oxide quantum dots and reduced graphene oxide quantum dots formed into freestanding pellets [55]. Their complex dielectric and conductivity spectra were analyzed using a Drude–Lorentz model, yielding a higher fitted carrier density but lower fitted mobility in the reduced samples. The authors associated the lower mobility with increased structural disorder indicated by Raman spectroscopy. These findings provide complementary context for interpreting the influence of chemical modification and disorder on the THz response of carbon-dot assemblies.

For the N-CND layer, THz magneto-optical measurements were performed at 80 K in the Faraday geometry described in Section 2.1, over approximately 0.2–1.2 THz and under magnetic fields from 0 to 8 T [4]. The transmitted THz time-domain waveforms and corresponding frequency-domain amplitude and phase spectra at 0 and 8 T are shown in Figure 5c,d, respectively. The transmitted amplitudes changed with magnetic field and differed between the +45° and −45° polarization angles at 8 T, whereas the corresponding phase spectra remained nearly coincident. Following Fourier transformation, the complex electric-field spectra measured at analyzer angles of +45° and −45° were combined to obtain the circular components using the procedure described in Section 2. The corresponding substrate-normalized circular transmission coefficients were converted into the complex sheet conductances, *G*_+_ (ω) and *G*_−_ (ω), using the thin-film relation introduced in Section 2.2.2.

As shown in Figure 5e, *G*_±_ (*ω*) coincided at zero magnetic field but separated progressively with increasing field, providing direct evidence of a finite magneto-optical response from the N-CND layer. At zero field, the real conductance increased with frequency, whereas the imaginary conductance decreased. At finite fields, both components of *G*_+_ (*ω*) increased with frequency; for *G*_−_ (*ω*), the real component decreased while the imaginary component increased. No obvious features associated with quantum-confined sub-bands or Landau quantization were observed in either the time- or frequency-domain spectra. Because the THz photon energy was substantially smaller than the electronic energy-level separations in the N-CNDs, the response was attributed to free-electron absorption rather than photon-induced carrier generation or excitonic effects. Moreover, because the THz wavelength was considerably larger than both the approximately 5.5 nm nanodots and the pores within the granular layer, the sample was treated as an effective uniform medium. The measured sheet-conductance spectra were then analyzed using the generalized magneto-optical Drude–Smith model described in Section 2.3.2 [4].

Simultaneous fitting of the real and imaginary components of both circular conductances yielded four quantities: the electron-density-related coefficient γ, relaxation time *τ*, effective electron mass m*, and electronic localization factor *α*. The coefficient γ is defined as γ = *gn*_e_, where *n*_e_ is the electron density and *g* is the geometric factor associated with the N-CND layer thickness. Because *g* could not be determined independently for the granular layer, the fitting yielded γ rather than the absolute electron density. The magnetic-field dependences of the four fitted quantities are summarized in Figure 5f. With increasing magnetic field, electron-density-related coefficient γ decreased from approximately 3.25 × 10^13^ cm^−2^ near zero field to 2.78 × 10^13^ cm^−2^ at 8 T. The study associated this field-dependent reduction with electronic localization under a strong magnetic field and a corresponding decrease in the electronic density of states [56]. In contrast, the relaxation time increased almost linearly from approximately 0.026 ps near zero field to 0.035 ps at 8 T. This increase was attributed to the decreasing radius of the cyclotron motion as the magnetic field increased, resulting in a reduction in the effective carrier-scattering rate [17]. Although *τ* increased with field, it remained short throughout the investigated range, indicating that strong carrier scattering persisted in the N-CND layer.

The electronic localization factor remained negative at all magnetic fields but became progressively less negative, changing from approximately −0.82 near zero field to −0.69 at 8 T. The resulting decrease in *α* indicates a weakening of electronic backscattering or localization. This behavior was attributed to magnetic-field-induced cyclotron motion, which reduces the probability of carrier backscattering [17,18]. Nevertheless, the localization factor remained far from the Drude limit of zero, demonstrating that restricted carrier motion persisted even at the highest magnetic field.

At zero field, the best fit was obtained using an effective electron mass of approximately 0.055 *m*_0_, slightly larger than the experimental bulk-graphite value of approximately 0.05 *m*_0_ [57]. This difference was attributed to nano-size effects, surface and edge states, defects, and chemical functional groups, which modify the electronic structure of N-CNDs relative to bulk graphite. At finite magnetic fields, m*/*m*_0_ was obtained from simultaneous fitting of the circular conductances and increased to approximately 0.063 *m*_0_ at 8 T [58]. The increase in m* with magnetic field was associated with magnetic confinement of the conducting electrons. On the other hand, no distinct cyclotron-resonance feature was resolved within the measured frequency range. At 8 T, the fitted effective mass of approximately 0.063 *m*_0_ corresponds to a cyclotron frequency *f*_c_ = *eB*/2πm* of approximately 3.55 THz, substantially above the experimental upper limit of 1.2 THz. Using the reported relaxation time of approximately 0.035 ps gives *ω*_c_*τ* ≈ 0.78 which is below the condition *ω*_c_*τ* > 1 required for a clearly resolved cyclotron resonance [47]. Consequently, the effective mass was determined by fitting the field-dependent circular conductances under a non-resonant condition.

Magneto-optical activity has also been observed in related carbon-dot systems at optical frequencies. Zhang et al. measured Faraday rotation in pyrolysis-synthesized graphene quantum dots and nitrogen-doped graphene quantum dots and found a pronounced dependence on particle structure and synthesis conditions [59]. The large rotation was associated primarily with extended sp^2^-hybridized electronic regions within the nanodots, while differences between the undoped and nitrogen-doped samples further indicated sensitivity to particle composition and structure. Because these measurements were performed in the visible spectral range, they probe different electronic processes from the low-energy intraband carrier response considered here. Nevertheless, they demonstrate that the magneto-optical response of carbon dots is strongly connected to their nanoscale electronic structure, complementing the THz magneto-optical carrier-dynamics picture obtained for N-CNDs.

Overall, the results establish a pronounced non-Drude magneto-optical response in the granular N-CND layer. The short relaxation time and strongly negative localization factor indicate substantial carrier scattering and preferential backscattering, while their magnetic-field dependences show a partial weakening of these effects. Although the N-CNDs have nanoscale dimensions, no discrete confinement-induced sub-bands were resolved in the measured THz range, and their low-energy response was described as that of a quasi-zero-dimensional electron gas. The study therefore demonstrates that THz magneto-optical spectroscopy can determine the relaxation, localization and effective-mass characteristics of dried N-CND layers without electrical contacts or a spectrally resolved cyclotron resonance.

## 4. Wide-Bandgap Materials

Wide-bandgap materials provide a distinct platform for THz magneto-optical spectroscopy because the energy of a THz photon is generally far below their fundamental bandgap [5,18]. Consequently, the measured low-energy response is not associated with intrinsic interband excitation but is governed primarily by free carriers introduced by dopants, vacancies, surface states and irradiation-induced defects. These extrinsic electronic states can produce measurable conductivity and magneto-optical effects even when the host material remains highly resistive. At the same time, impurity potentials, lattice disorder and defect complexes can strongly limit carrier motion, resulting in short relaxation times, pronounced backscattering and the absence of resolved cyclotron resonance within conventional THz frequency and magnetic-field ranges [60].

Nitrogen-doped diamond [18] and neutron-irradiated hexagonal boron nitride [5] are representative examples of this behavior. In nitrogen-doped diamond, nitrogen-related bonds, impurity states and vacancy centres modify the carrier density, dielectric response and effective electron mass. In neutron-irradiated hexagonal boron nitride, irradiation-induced defects alter the carrier population and relaxation dynamics. Unlike the atomically thin and granular systems discussed in Section 3, both materials were investigated as bulk specimens. Their magneto-optical responses were therefore analyzed through the circular refractive indices, dielectric functions and bulk conductivities rather than sheet conductances. These studies demonstrate how THz magneto-optical spectroscopy can quantify defect-mediated carrier transport in highly resistive wide-bandgap materials without requiring electrical contacts.

### 4.1. Nitrogen-Doped Diamond

**Figure 6:**
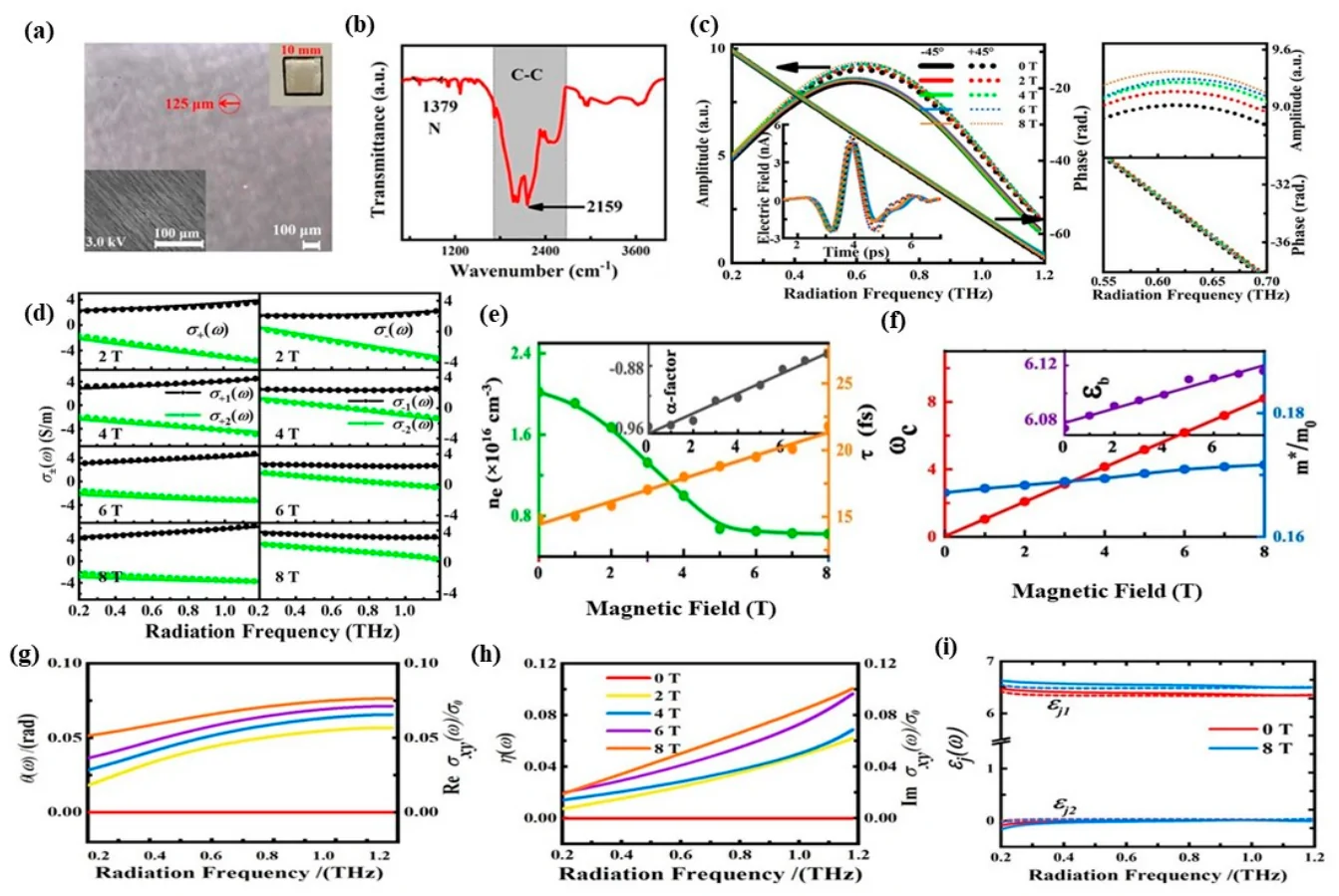
THz magneto-optical response of nitrogen-doped polycrystalline diamond. (**a**) Sample morphology showing an average grain size of approximately 125 μm; the insets show a photograph of the free-standing specimen and a scanning electron microscopy image. (**b**) FTIR spectrum showing nitrogen-related absorption and intrinsic diamond multiphonon absorption. (**c**) Transmitted THz amplitude and phase spectra measured at 80 K at polarizer orientations of −45° (solid lines) and +45° (dotted lines) under magnetic fields of 0–8 T. The inset shows the time-domain waveforms, and the plots on the right show enlarged spectral regions. (**d**) Real (black) and imaginary (green) components of the circular conductivities, *σ*_+_ (ω) and *σ*_−_ (ω), under magnetic fields of 2–8 T; symbols represent experimental results and curves represent generalized magneto-optical Drude–Smith fits. (**e**) Fitted electron density *n_e_* and relaxation time τ; the inset shows the localization factor *α*. (**f**) Cyclotron frequency *ω*_c_ and normalized effective mass m*/*m*_0_; the inset shows the background dielectric constant *ε*_b_. (**g**,**h**) Faraday rotation angle *θ* (ω) and ellipticity *η* (ω), together with the corresponding real and imaginary components of the normalized transverse conductivity, *σ*_xy_ (ω)/*σ*_0_. (**i**) Real and imaginary components of the circular dielectric functions at 0 and 8 T. Panels (**a**–**f**) adapted/reprinted with permission from Ref. [18]. Copyright © 2024 Elsevier B.V. Panels (**g**–**i**) reproduced from Ref. [68] under the CC BY-NC-ND license.

Diamond is a wide-bandgap semiconductor with a bandgap of approximately 5.5 eV, high thermal conductivity, broad optical transparency and excellent chemical and radiation stability [61,62]. The nitrogen-doped polycrystalline diamond investigated here was prepared by microwave plasma-enhanced chemical vapour deposition on silicon using methane and hydrogen with a small nitrogen addition, followed by annealing and substrate removal [63]. The free-standing specimen was approximately 1 mm thick, with an average grain size of approximately 125 μm; its morphology is shown in Figure 6a. X-ray diffraction confirmed the polycrystalline diamond structure, while Raman spectroscopy identified the characteristic diamond mode near 1331 cm^−1^ and an additional feature attributed to nitrogen-vacancy-related emission [64]. The FTIR spectrum as shown in Figure 6b exhibited intrinsic diamond multiphonon absorption and bands assigned to nitrogen-related bonding [65]. X-ray photoelectron spectroscopy further supported nitrogen incorporation through the observed N 1s components [66]. These measurements establish the structural and chemical context for the THz magneto-optical analysis.

The influence of nitrogen defects on carrier transport in diamond has also been demonstrated directly by time-resolved THz spectroscopy. Ulbricht et al. investigated photoexcited electrons in type-Ib synthetic diamond containing controlled concentrations of substitutional nitrogen and showed that electron mobility was limited by scattering from neutral substitutional nitrogen defects [67]. They further found that the subsequent electron recombination involved changes in the charge states of the nitrogen centres. Although these measurements probed optically excited nonequilibrium carriers rather than the equilibrium field-dependent response considered here, they establish that substitutional nitrogen can strongly influence carrier mobility and recombination dynamics in diamond. These findings provide relevant context for interpreting the THz magneto-optical carrier response of the nitrogen-doped polycrystalline diamond discussed below.

#### 4.1.1. Magneto-Optical Carrier Dynamics

The measurements were performed at 80 K using the polarization-resolved Faraday geometry described in Section 2.1, over an effective frequency range of approximately 0.2–1.2 THz and under magnetic fields from 0 to 8 T [18]. Figure 6c shows the transmitted frequency-domain amplitude and phase spectra measured at polarizer orientations of +45° and −45°, together with representative time-domain waveforms in the inset. The amplitude spectra exhibited magnetic-field and polarization dependence, whereas the phase spectra showed comparatively small differences. Following Fourier transformation, the complex electric-field spectra measured at the two orientations were combined to obtain the circular components using the procedure described in Section 2. Because the approximately 1-mm-thick specimen constituted a bulk slab, its circular transmission coefficients were analyzed using the bulk-sample formalism introduced in Section 2.2 to obtain the complex refractive indices, dielectric functions and magneto-optical conductivities.

As shown in Figure 6d, the two circular conductivities developed increasingly different spectral responses with increasing magnetic field. For *σ*_+_ (*ω*), the real component increased with field, whereas the imaginary component remained negative and decreased. The real part of *σ*_−_ (*ω*) initially decreased and subsequently increased, while its imaginary component increased and changed sign depending on frequency and magnetic field. The increasing difference between the circular conductivities reflects the field-induced magneto-optical response, although no distinct cyclotron-resonance feature was resolved. These spectra were analyzed using the generalized magneto-optical Drude–Smith model described in Section 2.3.2 [10]. Simultaneous fitting of the circular dielectric functions and conductivities yielded the static dielectric constant *ϵ*_b_, electron density *n*_e_, relaxation time *τ*, electronic localization factor *α*, cyclotron frequency *ω*_c_ and effective electron mass m*. Their magnetic-field dependences are summarized in Figure 6e,f.

The fitted electron density decreased from approximately 2.0 × 10^16^ cm^−3^ near zero field to approximately 0.6 × 10^16^ cm^−3^ at 8 T. Although this density is low compared with that of conventional doped semiconductors, it is substantially higher than the value of approximately 10^14^ cm^−3^ previously reported for nominally undoped single-crystal diamond [69]. The higher density is therefore consistent with an increased electron contribution following nitrogen incorporation. The authors associated the field-dependent reduction in *n*_e_ with electronic localization, charged-impurity scattering and a magnetic-field-induced decrease in the electronic density of states [56,70]. Because *n*_e_ was obtained through model fitting, it should be interpreted as the effective electron population contributing to the measured THz response. In contrast, the relaxation time increased from approximately 14–15 fs near zero field to approximately 21–22 fs at 8 T. This increase was attributed to magnetic-field-induced cyclotron motion, which reduces the effective interaction of conducting electrons with scattering centres such as impurities and phonons [2]. Nevertheless, the relaxation time remained extremely short throughout the investigated field range, demonstrating that strong carrier scattering persisted even at 8 T.

Moreover, using the relation *μ* = *eτ*/m*, the authors estimated a scattering-time mobility of approximately 200 cm^2^V^−1^s^−1^ near zero field at 80 K [18]. This quantity does not include the suppression of long-range transport represented by the electronic localization factor. In the zero-field Drude–Smith limit, the corresponding dc mobility is given by *μ_dc_* = *eτ*/m* (1 + α). The near-zero-field value of α ≈ −0.96 therefore reduces the dc mobility to only a small fraction of the scattering-time mobility, emphasizing the substantial suppression of long-range carrier transport by preferential backscattering. The localization factor remained strongly negative throughout the investigated magnetic-field range but became progressively less negative, changing from approximately −0.96 near zero field to approximately −0.86 at 8 T as shown in Figure 6e. The corresponding decrease in |*α|* indicates a modest weakening of electronic backscattering or localization. The authors attributed this trend to cyclotron motion under magnetic field, which reduces the probability of electron backscattering [17,18]. However, because *α* remained close to −1, the electronic response was still far from the unrestricted Drude limit even at the highest field.

The fitted static dielectric constant increased only slightly, from approximately 6.08 near zero field to approximately 6.12 at 8 T as illustrated in Figure 6f. The zero-field value corresponds to a refractive index of approximately 2.47, consistent with previously reported THz values for diamond [71]. The authors associated the slightly enhanced dielectric constant relative to undoped single-crystal diamond with the additional polarizability introduced by nitrogen-related bonds and functional groups. Its weak magnetic-field dependence was attributed to further polarization of the corresponding electric dipoles, although the small magnitude of the variation warrants cautious interpretation [72].

The cyclotron frequency increased approximately linearly with magnetic field, consistent with the relation *ω*_c_ = eB/m*. The fitted effective electron mass was approximately 0.167 *m*_0_ near zero field and increased slightly toward 0.172 *m*_0_ at 8 T [58] as displayed in Figure 6f. The near-zero-field value was compared with the theoretical effective mass of approximately 0.15 *m*_0_ reported for electrons in the (111) plane of pure diamond [73]. The modest mass enhancement was associated with nitrogen-induced modification of the electronic structure, whereas the weak field dependence was attributed to magnetic confinement. These fitted values should be interpreted as effective transport masses within the magneto-optical Drude–Smith description rather than as a complete representation of the anisotropic conduction-band masses of diamond.

No resolved cyclotron-resonance peak was detected between 0.2 and 1.2 THz. At 8 T, the fitted effective mass gives a cyclotron angular frequency of approximately 8.2 × 10^12^ rad s^−1^, corresponding to a cyclotron frequency of *f*_c_ = *ω*/2π ≈ 1.30 THz. This frequency lies close to or slightly above the upper limit of the reliable experimental window. More importantly, the short relaxation time gives *ω*_c_*τ* ≈ 0.17–0.18, substantially below the condition *ω*_c_*τ* > 1 required for a well-resolved cyclotron resonance [2,17]. Assuming that the fitted effective mass and relaxation time remain approximately unchanged, a magnetic field of about 48.4 T would be required to reach *ω*_c_*τ* ≈ 1, with the corresponding resonance expected near 8 THz. The absence of cyclotron resonance was therefore governed primarily by the short relaxation time and low carrier mobility rather than by the available spectral bandwidth alone.

The absence of a resolved cyclotron resonance in nitrogen-doped polycrystalline diamond contrasts with the response reported for ultrapure single-crystal diamond. Naka et al. employed time-resolved cyclotron resonance of optically excited carriers to determine anisotropic electron and hole effective masses [74]. The resonance linewidths indicated scattering times exceeding 100 ps and carrier mobilities approaching 10^6^ cm^2^ V^−1^ s^−1^ at 10 K. These scattering times are substantially longer than the relaxation times of tens of femtoseconds extracted for the nitrogen-doped polycrystalline sample. Although the samples and measurement conditions differ, the comparison illustrates the importance of carrier scattering in determining whether cyclotron resonance can be resolved.

Despite the non-resonant measurement conditions, analysis of the circular spectra provided model-based estimates of the carrier density, relaxation time, localization factor, static dielectric constant and effective mass. The short relaxation time and strongly negative localization factor indicate substantial scattering and preferential backscattering in the nitrogen-doped polycrystalline sample.

#### 4.1.2. Faraday Rotation and Ellipticity

A complementary polarization-resolved THz study examined the Faraday rotation and ellipticity of nitrogen-doped diamond at 80 K under magnetic fields from 0 to 8 T [68]. These quantities were evaluated using the relations presented in Section 2.2.4. At zero field, the two circular components experienced nearly identical amplitude and phase responses, resulting in negligible rotation and ellipticity. Applying a magnetic field removed this equivalence and produced finite differences between their complex transmission coefficients. As shown in Figure 6g, the Faraday rotation increased systematically with both magnetic field and frequency, reaching approximately 0.05 rad (2.9°) at 0.2 THz and 0.077 rad (4.4°) at 1.2 THz under 8 T. The ellipticity exhibited a similar field dependence but varied more strongly with frequency, increasing from approximately 0.02 to 0.10 over the same spectral range as shown in Figure 6h. Their smooth frequency dependences, rather than resonant line shapes, are consistent with the absence of a resolved cyclotron resonance [21].

The polarization response was also expressed through the normalized complex transverse conductivity *σ*_xy_(*ω*)/*σ*_0_. Its real and imaginary components followed the respective frequency dependences of the Faraday rotation and ellipticity as shown in Figure 6g,h [68], linking the measured polarization changes to the magnetic-field-induced off-diagonal conductivity response. The corresponding circular dielectric functions are shown in Figure 6i. Their real components remained near 6.5 and exhibited only weak frequency dependence, whereas the imaginary components were considerably smaller. The two circular dielectric functions were nearly identical at zero field but developed a small separation at 8 T, indicating different phase velocities and attenuation coefficients for the two circular eigenmodes. These frequency-dependent dielectric functions include both background polarization and free-carrier contributions and should therefore be distinguished from the fitted parameter *ϵ*_b_ discussed in Section 4.1.1, which represents only the background contribution used in the magneto-optical Drude–Smith analysis.

Although no cyclotron-resonance feature was resolved, the difference between the two circular responses produced appreciable broadband THz polarization rotation under non-resonant conditions. These results demonstrate the potential of nitrogen-doped diamond for magnetic-field-controlled THz polarization modulation [68]. However, the reported response was obtained at 80 K and under fields approaching 8 T. Operation at higher temperatures and lower magnetic fields, together with control of the accompanying ellipticity and absorption, would improve its practical applicability.

### 4.2. Neutron-Irradiated Hexagonal Boron Nitride

Hexagonal boron nitride (hBN) is an ultrawide-bandgap semiconductor with a bandgap of approximately 6 eV, together with high thermal stability, chemical inertness, mechanical strength and resistance to oxidation and nuclear radiation [75]. Its constituent elements have low atomic numbers, resulting in relatively weak sensitivity to γ-ray radiation, whereas the ^10^*B* isotope has a large thermal-neutron-capture cross-section [76]. This combination makes hBN an attractive material for solid-state neutron detection. Because the probability of neutron capture increases with active-material thickness, large-area and millimetre-thick hBN samples are particularly desirable. However, producing single-crystalline hBN with these dimensions remains technically difficult and expensive. Farooq et al. therefore investigated polycrystalline pyrolytic boron nitride (PBN) as a scalable neutron-sensitive material and used THz magneto-optical spectroscopy to determine how neutron irradiation modifies its carrier transport and dielectric response [5].

**Figure 7:**
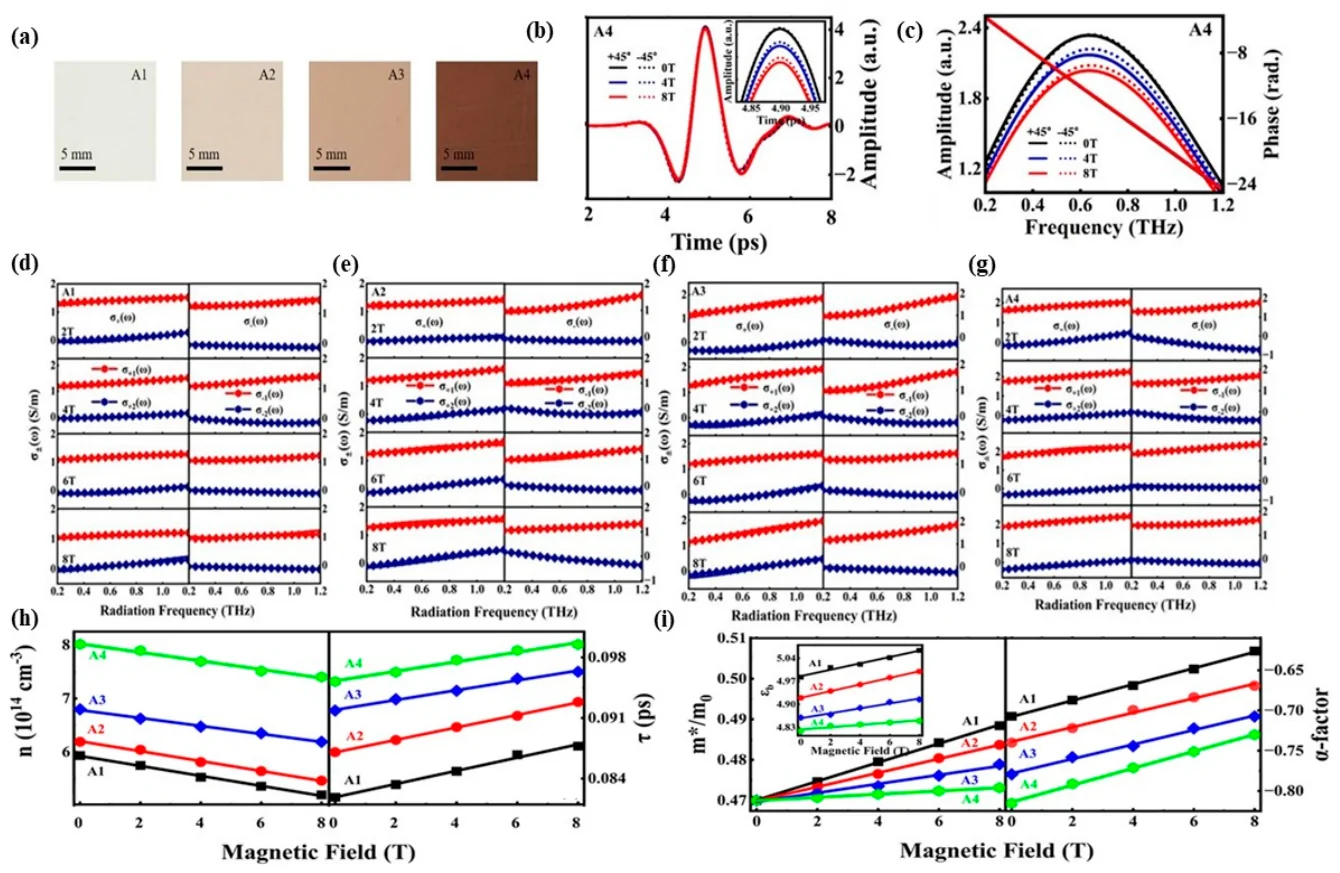
THz magneto-optical response of neutron-irradiated pyrolytic boron nitride. (**a**) Photographs of the unirradiated sample A1 and samples A2–A4 exposed to increasing neutron fluence, as specified in Table 1. (**b**) Representative transmitted THz time-domain waveforms for A4; the inset highlights the waveform maxima. (**c**) Corresponding frequency-domain amplitude and phase spectra. (**d**–**g**) Real (red) and imaginary (blue) components of the circular conductivities, *σ*_+_ (ω) and *σ*_−_ (ω), for A1–A4, respectively, measured at 80 K under magnetic fields of 2–8 T. Symbols represent experimental data and curves represent generalized magneto-optical Drude–Smith fits. (**h**) Magnetic-field dependence of the fitted hole density *n* and relaxation time *τ*. (**i**) Normalized effective hole mass m*/*m*_0_ and localization factor *α*; the inset shows the static dielectric constant *ε*_b_. Adapted from Ref. [5] under Elsevier author reuse rights. Copyright © 2025 Elsevier B.V.

The PBN samples were prepared by high-temperature chemical vapour deposition using borazine, with nitrogen as the carrier and dilution gas [77]. The free-standing specimens had lateral dimensions of approximately 14 × 14 mm^2^ and a thickness of approximately 1 mm. TEM and SEM measurements revealed locally ordered hBN crystallites assembled into stacked lamellae, with crystallite boundaries, folds and interlamellar interfaces providing a heterogeneous environment for carrier transport [77]. The unirradiated sample was designated A1, while A2–A4 received progressively higher thermal-neutron fluences, as summarized in Table 1. The samples were irradiated from multiple directions to reduce directional nonuniformity. Their progressive color change from white for A1 to light red, red and deep red for A2, A3 and A4, respectively, is shown in Figure 7a and provides visual evidence of irradiation-induced modification.

Neutron capture by ^10^B produces energetic lithium ions and *α* particles that generate displacement damage and defect centres in the surrounding lattice [78,79]. The colour evolution and previously reported photoluminescence changes were associated with irradiation-induced modification of defect-related electronic states [77]. Consistent with this interpretation, Toledo et al. detected neutron-induced EPR-active point defects in hBN and tentatively assigned the dominant centre to doubly occupied nitrogen vacancies with S = 1 [80]. Because the estimated thermal-neutron absorption length of approximately 277 μm is smaller than the sample thickness [81], the irradiation-induced modification is nonuniform through the specimen. The THz measurements therefore yield effective optical and transport parameters for the probed inhomogeneous volume rather than spatially resolved properties of the irradiated regions.

The electronic response of the PBN samples was measured at 80 K over approximately 0.2–1.2 THz under magnetic fields from 0 to 8 T using the Faraday geometry described in Section 2.1 [5]. Representative transmitted time-domain waveforms and corresponding frequency-domain amplitude and phase spectra for A4 are shown in Figure 7b,c. The amplitude spectra exhibited field- and polarization-dependent differences, whereas the phase spectra remained nearly coincident. Following Fourier transformation, the complex electric-field spectra measured at polarizer orientations of +45° and −45° were combined to obtain the circular components using the procedure described in Section 2. Because the samples were approximately 1 mm thick, the bulk optical formalism introduced in Section 2.2 was used to obtain the complex circular refractive indices, dielectric functions and magneto-optical conductivities.

At zero magnetic field, the two circular conductivities overlapped within the experimental uncertainty and developed increasingly different responses with increasing field as shown in Figure 7d–g. For *σ*_+_ (ω), both the real and imaginary components increased with frequency, whereas for *σ*_−_ (ω), the real component increased and the imaginary component decreased. No distinct cyclotron-resonance peak was resolved. The spectra were fitted using the generalized magneto-optical Drude–Smith model described in Section 2.3.2 [5,10], yielding the effective hole density *n*, relaxation time τ, localization factor *α*, static dielectric constant *ε*_b_ and cyclotron frequency *ω*_c_, from which the finite-field effective mass (m*) was determined.

The PBN samples were treated as p-type, consistent with previous reports on CVD-grown PBN [77]. Their mobile holes were associated primarily with the ionization of nitrogen-vacancy-related centers, although background impurities, boron vacancies and other defects may also contribute. The fitted zero-field hole density increased systematically with neutron fluence, rising from approximately 5.9 × 10^14^ cm^−3^ for the unirradiated A1 sample to 6.2 × 10^14^, 6.8 × 10^14^ and 8.0 × 10^14^ cm^−3^ for A2, A3 and A4, respectively, as shown in Figure 7h [5]. This increase was associated with additional vacancy-related centres and electrically active defects generated by neutron irradiation [60,82]. Because *n* was determined through optical-model fitting, it represents the effective hole population contributing to the measured THz response rather than the total density of irradiation-induced defects. Moreover, the zero-field relaxation time also increased with neutron fluence, from approximately 0.082 ps for A1 to approximately 0.095 ps for A4 as displayed in Figure 7h. Neutron irradiation introduces additional charged defects that can enhance carrier-defect scattering, but it can also generate trapping centres and modify carrier–phonon coupling. Previous Raman measurements indicated a reduction in carrier–phonon scattering with increasing neutron fluence, which was associated with irradiation-induced lattice distortion [77]. The observed increase in *τ* therefore reflects the combined influence of several competing scattering and trapping processes rather than a simple reduction in the total structural disorder [60].

Despite the increases in *n* and *τ*, the electronic localization factor became more negative with increasing neutron fluence. Near zero field, *α* changed from approximately −0.71 for A1 to approximately −0.81 for A4 as shown in Figure 7i. The corresponding increase in |*α|* indicates stronger electronic localization following neutron irradiation. This trend was attributed to irradiation-induced vacancies, defect complexes and lattice distortion, which move the polycrystalline PBN response further from the conventional Drude limit. Konusov et al. similarly showed that carbon-ion irradiation modified defect states within the bandgap of pyrolytic boron nitride and produced low-conducting surface layers in which transport involved donor- and acceptor-like levels associated with radiation and growth defects [83]. Although the irradiation conditions differ from those considered here, these results demonstrate that radiation-induced defect states can substantially modify carrier-transport pathways in PBN. In the neutron-irradiated samples considered here, the increase in the fitted hole population therefore occurs concurrently with stronger restriction of long-range carrier motion [60].

The static dielectric constant also depended on neutron fluence. Near zero field, *ϵ*_b_ decreased from approximately 4.97 for A1 to approximately 4.82 for A4 as displayed in the inset of Figure 7i. This reduction was associated with the disruption of lattice-related polarization by irradiation-induced nitrogen and boron vacancies, grain boundaries and local lattice distortion [78,80]. In polycrystalline PBN, these structural imperfections reduce the effective electric-dipole response relative to that of high-quality single-crystal hBN [60]. The fitted *ϵ*_b_ represents the background contribution used in the magneto-optical transport model and should be distinguished from the total frequency-dependent circular dielectric functions.

Application of a magnetic field produced systematic changes in all fitted parameters. For each sample, the effective hole density decreased with increasing field as shown in Figure 7h. At 8 T, *n* reached approximately 5.1 × 10^14^, 5.5 × 10^14^, 6.2 × 10^14^ and 7.3 × 10^14^ cm^−3^ for A1, A2, A3 and A4, respectively. The authors attributed this reduction to two main mechanisms. First, the magnetic field can increase the binding energy of impurity- and defect-derived states, thereby reducing the ionization of nitrogen-vacancy-related centres [5]. Second, the reduction in cyclotron radius and the associated field-induced localization can modify the carrier density of states [70]. The fitted decrease in *n* was therefore interpreted as a characteristic magnetic-field response of the defect-mediated hole population rather than as a change produced by neutron fluence alone. In contrast, the relaxation time increased moderately with magnetic field, reaching approximately 0.087–0.099 ps at 8 T across A1–A4 as shown in Figure 7h [5]. This increase was attributed to the confinement of carrier motion within cyclotron trajectories before the carriers interact with defects, impurities or phonons, thereby reducing the effective scattering rate [2,17]. The field-induced increase in *τ* occurred in all four samples.

The localization factor simultaneously became less negative with increasing magnetic field. At 8 T, *α* ranged from approximately −0.63 for A1 to approximately −0.75 for A4 as shown in Figure 7i. The resulting decrease in |*α|* indicates weaker preferential backscattering among the carriers contributing to the THz response. This behavior was associated with cyclotron motion, which reduces the probability that a carrier reverses its velocity after interacting with a scattering centre [18]. Nevertheless, the localization factor remained strongly negative for all samples, showing that restricted carrier motion persisted even at 8 T. Moreover, the systematically more negative values for the irradiated samples demonstrate that the magnetic field only partially counteracted the enhanced localization introduced by neutron irradiation.

The static dielectric constant increased slightly with magnetic field for every sample, reaching values of approximately 4.85–5.06 at 8 T as shown in the inset of Figure 7i. The authors attributed this weak increase to further polarization of the effective electric dipoles under magnetic field. Across the complete dataset, however, the neutron-fluence dependence remained evident: at a fixed magnetic field, *ϵ*_b_ decreased systematically from A1 to A4. At zero field, an effective hole mass of 0.47 *m*_0_ was adopted as an input parameter for all four samples [60]. At finite magnetic fields, the cyclotron frequency was treated as a fitting parameter, allowing m* to be determined from *ω*_c_ = *eB*/m*. The fitted effective mass increased with magnetic field for each sample and reached approximately 0.47–0.51 *m*_0_ at 8 T as shown in Figure 7i. The authors associated this increase with magnetic confinement of the carrier motion. At a fixed finite field, m* decreased slightly as the neutron fluence increased, a trend attributed to irradiation-induced lattice distortion [60,77]. Because these masses were extracted from a strongly damped, non-resonant response, they should be interpreted as effective transport masses describing defect-mediated hole motion rather than as intrinsic valence-band masses of ideal hBN.

No distinct cyclotron-resonance feature was resolved within the measured spectral range [5]. Using the fitted masses and relaxation times at 8 T gives cyclotron frequencies of approximately 0.44–0.48 THz and values of *ω*_c_τ of only about 0.24–0.29. Although the corresponding cyclotron frequencies lie within the experimental window, the condition *ω*_c_τ > 1 required for a well-resolved resonance was not satisfied. The absence of cyclotron resonance was therefore governed primarily by the relatively heavy effective hole mass and strong collision-induced damping. Nevertheless, simultaneous analysis of the two circular channels provided sufficient field-dependent information to determine the cyclotron frequency and effective mass under non-resonant conditions.

Overall, neutron irradiation produced competing modifications of the electronic response of PBN [60,77]. Increasing fluence raised the effective hole density and relaxation time but strengthened preferential backscattering and reduced the static dielectric constant. Application of a magnetic field reduced the fitted hole density while increasing the relaxation time, weakening preferential backscattering and slightly enhancing the static dielectric response. These results show that irradiation-induced carrier generation does not necessarily correspond to improved long-range transport because the same irradiation process also creates defects and lattice distortion that enhance localization. This competition between carrier generation and restricted carrier motion is an important consideration when evaluating thick PBN for neutron-sensitive electronic and optoelectronic applications, although the THz measurements do not directly determine the charge-collection efficiency of a complete neutron-detector device [5].

## 5. Cross-Material Trends and Limitations

The studies reviewed in Section 3 and Section 4 demonstrate the applicability of THz magneto-optical spectroscopy to atomically thin layers, granular nanostructures and bulk wide-bandgap materials. Quantitative comparison among these systems requires consideration of the different electrodynamic quantities used to describe their responses. Monolayer and bilayer MoS_2_ were analyzed using thin-film conductivities and areal electron densities [16,17], whereas N-doped diamond and neutron-irradiated PBN were treated as bulk media and characterized by bulk conductivities, dielectric functions and volume carrier concentrations [5,18]. For N-CNDs, the uncertain active thickness of the granular layer prevented independent determination of the absolute electron concentration; fitting of the sheet conductance therefore yielded the density-related coefficient γ = *gn*_e_ [4]. Moreover, the parameters obtained for PBN represent effective averages over the nonuniform irradiation profile across the sample thickness. Absolute carrier-density values are consequently not directly comparable among these materials [5]. Their measured responses, density definitions and principal parameter trends are summarized in Table 2.

Across the investigated systems, the generalized magneto-optical Drude–Smith analysis yields recurring field-dependent trends, including a decrease in the density-related quantity, an increase in relaxation time, and a reduction in the magnitude of the backscattering parameter [4,5,16,17,18]. An increase in effective mass was also obtained in the systems where it was treated as a fitting parameter [4,5,17,18]. The physical interpretation of these fitted trends depends on the characteristics of each material system. Changes in carrier scattering associated with cyclotron motion, magnetic-field-induced localization, changes in the electronic density of states, and magnetic confinement have been considered in interpreting the observed field dependence. For PBN, enhanced defect binding and reduced ionization have also been proposed to account for the decrease in the fitted hole density. Importantly, the magnetic-field-induced changes in the complex THz response are directly observed, whereas the individual carrier parameters are obtained through simultaneous fitting. Because these parameters jointly determine the calculated response, correlations among them may influence their extracted field dependences. Similar fitted trends may therefore have different physical origins in different material systems.

A reduction in *n* or γ represents a decrease in the effective carrier-density contribution required to reproduce the THz response and does not necessarily indicate an equivalent decrease in the total carrier population. Similarly, *α* describe preferential backscattering within the Drude–Smith model but do not directly measure a localization length, defect concentration or universal degree of disorder. Their absolute values are therefore not directly comparable among atomically thin, granular and bulk polycrystalline systems.

Polarization-resolved measurements provide stronger constraints on cyclotron dynamics than measurements of longitudinal conductivity alone. Simultaneous analysis of the real and imaginary components of the LHCP and RHCP responses enabled the cyclotron frequency and effective mass to be obtained for bilayer MoS_2_, N-CNDs, N-doped diamond and PBN [4,5,17,18]. In monolayer MoS_2_, only the longitudinal conductivity was measured and m* = 0.39 *m*_0_ was fixed during fitting; the other extracted carrier parameters are therefore conditional on the assumed mass [16]. None of the five principal systems summarized in Table 2 exhibited a resolved cyclotron-resonance peak. For N-CNDs, the fitted cyclotron frequency at 8 T lay above the measured spectral range, while the short relaxation time further broadened the response [4]. In N-doped diamond and PBN, collision-induced damping prevented the condition *ω*_c_τ > 1 from being satisfied [5,18]. The relatively large effective masses and finite relaxation times similarly suppressed the resonance in MoS_2_ [16,17]. The masses obtained from these measurements should therefore be regarded as effective transport masses extracted under non-resonant conditions.

Despite these limitations, all five principal systems exhibited clear magnetic-field-induced changes in their complex THz responses, and N-doped diamond additionally showed measurable Faraday rotation and ellipticity without a resolved cyclotron resonance [68]. These observations demonstrate that a resolved resonance is not essential for investigating field-dependent carrier dynamics, although it would provide a more direct determination of effective mass and reduce reliance on multiparameter fitting. THz magneto-optical spectroscopy therefore offers a common contact-free framework for comparing carrier dynamics across different material classes, provided that the extracted parameters are interpreted in relation to sample geometry, spectral bandwidth, carrier damping and the assumptions of the adopted model.

## 6. Outlook

**Figure 8:**
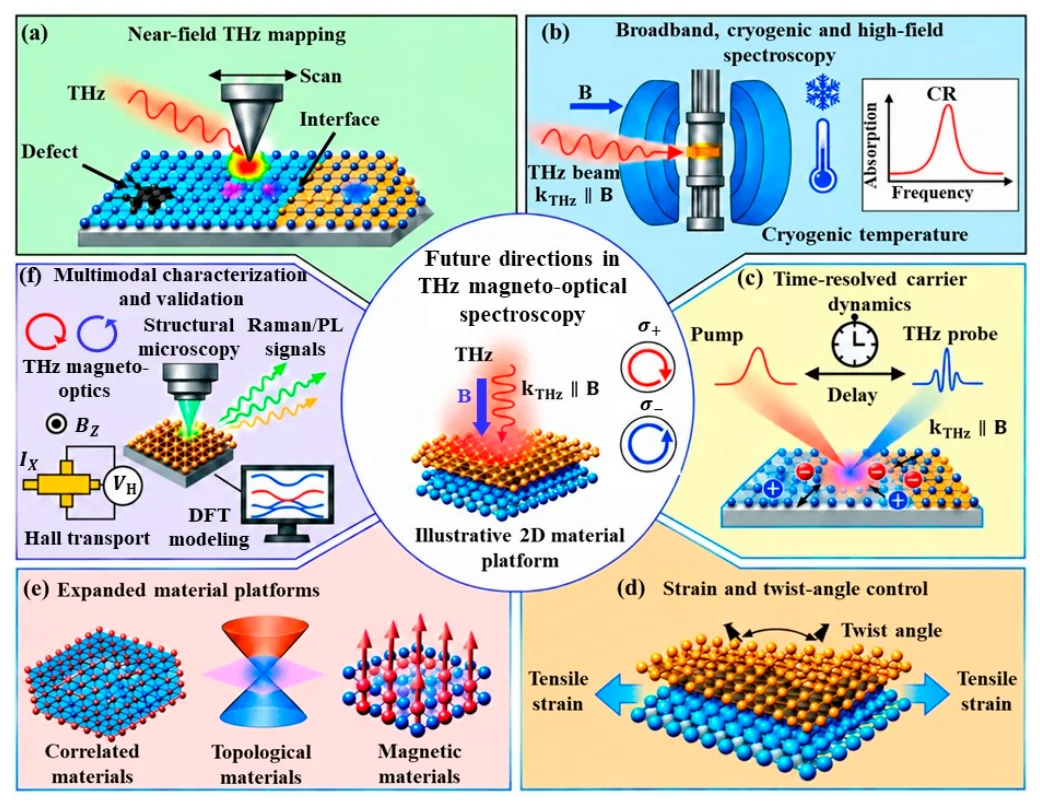
Future directions and emerging opportunities in THz magneto-optical spectroscopy. The central schematic illustrates an exemplary two-dimensional material platform probed in the Faraday geometry, with the THz propagation direction parallel to the magnetic field and the magneto-optical response resolved into two circular conductivity channels. The surrounding panels highlight key research directions: (**a**) spatially resolved near-field THz mapping of defects, interfaces, and electronic inhomogeneity; (**b**) broadband, cryogenic, and high-field spectroscopy for resolving cyclotron resonance and other field-induced excitations; (**c**) optical-pump-THz-probe measurements under an external magnetic field for tracking time-resolved carrier dynamics; (**d**) strain and twist-angle control of the electronic structure in layered materials; (**e**) expansion to correlated, topological, and magnetic material platforms; and (**f**) multimodal characterization and validation combining THz magneto-optical spectroscopy with Hall transport, structural microscopy, Raman and photoluminescence spectroscopy, and electronic-structure calculations.

The cross-material comparison in Section 5 identifies a common challenge for future THz magneto-optical studies: obtaining carrier parameters with less dependence on non-resonant multiparameter fitting. In the reviewed studies, measurements were generally limited to approximately 0.2–1.2 THz, magnetic fields up to 8 T and temperatures near 80 K [4,5,16,17,18]. None of the systems exhibited a resolved cyclotron-resonance peak. This absence did not arise from short relaxation times alone; depending on the material, it reflected a combination of limited spectral coverage, small *ω*_c_τ, relatively large effective mass and strong non-Drude damping. Consequently, parameters such as the cyclotron frequency and effective mass were inferred from field-induced changes in the complex spectra rather than determined directly from a resonance. The principal opportunities for addressing these limitations are summarized schematically in Figure 8.

Improved instrumentation should address spatial inhomogeneity and direct access to cyclotron dynamics as complementary objectives as illustrated in Figure 8a,b. Near-field THz microscopy could provide subwavelength maps of the carrier response and identify electrically heterogeneous regions, interfaces and irradiation-induced nonuniformity that are averaged in conventional far-field measurements as shown in Figure 8a [84]. Measurements over broader frequency ranges and at higher magnetic fields would increase the likelihood of directly resolving cyclotron resonance as illustrated in Figure 8b. For approximately unchanged m* and τ, increasing the magnetic field raises both *ω*_c_ and *ω*_c_τ; however, it also shifts the resonance toward higher frequencies, requiring the spectral bandwidth to be extended simultaneously. High-field THz measurements using pulsed magnetic fields up to 31 T have demonstrated that resolved cyclotron resonance can provide direct values of effective mass, density and scattering time [85]. For N-CNDs, spectral coverage extending beyond approximately 3.6 THz would be required to access the fitted cyclotron frequency at 8 T, which lies well above the experimental upper limit of 1.2 THz [4]. Even with this extended bandwidth, resolving the resonance may require a larger *ω*_c_τ, because its fitted value remained below unity. For bilayer MoS_2_, nitrogen-doped diamond and PBN, stronger fields could improve *ω*_c_τ, although reducing collision-induced damping through improved sample quality would also be important. Under cryogenic and high-field conditions, polarization-resolved THz measurements could additionally be extended toward quantum Hall and topological magneto-optical phenomena [86,87].

Time-resolved measurements provide further constraints on the extracted transport parameters, as shown in Figure 8c. Carrier-density control offers a complementary route for testing the fitted parameters. Most systems reviewed here were investigated only near 80 K, where impurity-, defect-, interface- and phonon-related scattering may contribute simultaneously. Measurements over a broader temperature range could help separate these contributions and determine whether the observed field dependences persist toward room temperature. Electrostatic gating could tune the carrier density and Fermi level in thin-film materials and has already been used to control THz magneto-optical responses in graphene [14]. Controlled doping or photoexcitation could provide complementary approaches for granular and bulk samples. Combining optical-pump-THz-probe spectroscopy with polarization-resolved detection and an external magnetic field would allow the transient circular response to be followed after excitation. High-field optical-pump-THz-probe measurements have already demonstrated time-resolved detection of cyclotron resonance and carrier-dependent relaxation dynamics [88]. Applied to the materials reviewed here, this approach could investigate intervalley relaxation in MoS_2_, intradot and interdot motion in N-CND films, and carrier trapping by dopant- or irradiation-induced states in diamond and PBN. Global analysis over pump delay, temperature, magnetic field and carrier density would be necessary to distinguish changes in carrier population from changes in scattering and localization.

Controlled modification of material structure provides another route for connecting the THz response with microscopic carrier environments as illustrated in Figure 8d. For layered materials, systematic variation in layer number, twist angle, strain and dielectric environment could clarify the effects of interlayer coupling, valley occupation and interface disorder. Twist-angle control has revealed correlated electronic phases in transition-metal–dichalcogenide bilayers [89], while biaxial strain can substantially modify the electronic structure of MoS_2_ [90]. For N-CND films, accurate measurements of active thickness, mass loading, porosity and packing density are required to separate geometric contributions from intrinsic carrier dynamics. Systematic variation in nanodot size, nitrogen content and surface chemistry could then help distinguish carrier motion within individual dots from transport through the assembled network [4]. For nitrogen-doped diamond and PBN, the fitted carrier parameters should be correlated with independently measured dopant concentrations, grain structures, irradiation fluences and defect populations [5,18]. The material scope could also be expanded to correlated, topological and magnetic systems in which THz magneto-optical spectroscopy may probe collective, Hall-like and magnetically active excitations as shown in Figure 8e [86,87]. The twisted-bilayer WSe_2_ device shown at the centre of Figure 8 should be regarded as an illustrative future platform rather than one of the systems reviewed in Section 3 and Section 4.

Further progress requires material-specific modeling and independent validation of the extracted parameters. Although the magneto-optical Drude–Smith model reproduced the non-Drude spectra considered in this review, its localization factor does not uniquely identify the microscopic origin of the suppressed low-frequency conductivity. A Drude–Smith-like response may also arise from a diffusive restoring current rather than exclusively from persistent carrier backscattering [91]. Effective-medium or network models should therefore be evaluated for granular N-CND layers, depth-dependent optical models for neutron-irradiated PBN and multivalley transport models for MoS_2_. Future fitting should report parameter uncertainties, parameter correlations and comparisons among alternative models. Combining THz spectroscopy with Hall measurements, Raman and photoluminescence spectroscopy, microscopy and electronic-structure calculations would provide independent tests of the fitted carrier density, relaxation time, localization factor and effective mass [4,5,16,17,18]. Beyond this validation, polarization-resolved THz spectroscopy could form part of a broader multimodal framework for studying charge, spin and valley dynamics, as illustrated in Figure 8f.

Beyond fundamental investigations of carrier dynamics, THz magneto-optical spectroscopy offers opportunities for contact-free material assessment and polarization control. For neutron-sensitive PBN, the THz-derived carrier parameters should be compared with device-level quantities such as charge-collection efficiency, leakage current and neutron-detection efficiency, which are commonly used to evaluate BN-based neutron detectors [92]. Materials exhibiting appreciable Faraday rotation or circular dichroism could also be explored for THz modulators, isolators and other nonreciprocal polarization components. Large and electrically tunable THz magneto-optical responses have already been demonstrated in graphene [14,93], while nitrogen-doped diamond exhibits measurable Faraday rotation under non-resonant conditions [68]. Practical implementation will nevertheless require significant rotation or modulation at higher temperatures and lower magnetic fields while maintaining adequate transmission, controlled ellipticity, broad operating bandwidth and thermal stability. Realizing these opportunities will require coordinated advances in instrumentation, sample design, material-specific modeling and independent experimental validation.

## 7. Conclusions

THz magneto-optical spectroscopy provides a contact-free approach for investigating carrier transport in materials for which reliable electrical contacts or conventional magneto transport measurements are difficult to realize. This review has considered representative systems spanning monolayer and bilayer MoS_2_ [16,17], granular N-CND films [4], nitrogen-doped diamond [18] and neutron-irradiated PBN [5]. Despite substantial differences in dimensionality, electronic structure, sample geometry and disorder, these materials can be examined within a common experimental framework based on THz time-domain spectroscopy in the Faraday geometry. Appropriate thin-film, sheet-conductance and bulk optical formalisms allow the measured longitudinal or circular responses to be related to the electrodynamic properties of each sample class.

In the polarization-resolved studies, the magnetic-field-induced separation of the two circular responses provided direct sensitivity to cyclotron dynamics. Simultaneous analysis of their real and imaginary components using the magneto-optical Drude–Smith model yielded the carrier-density-related contribution, relaxation time, localization factor and cyclotron frequency, from which an effective transport mass was inferred. For bulk samples, the static dielectric constant was also included in the analysis. Monolayer MoS_2_ represents a distinct case because only the longitudinal conductivity was analyzed, and the effective mass was fixed during fitting. None of the reviewed systems exhibited a resolved cyclotron-resonance peak; consequently, the fitted masses should be regarded as model-dependent effective transport masses rather than direct measurements of intrinsic band-edge masses. Their agreement with reported theoretical estimates for bilayer MoS_2_ and nitrogen-doped diamond nevertheless supports the internal consistency of the analysis.

A common non-Drude response emerged across the reviewed materials. With increasing magnetic field, the fitted density-related quantity generally decreased, the relaxation time increased and the magnitude of the photoinduced electronic localization factor decreased, indicating weaker preferential backscattering within the adopted model. These quantities were obtained simultaneously and jointly determine the measured spectra; therefore, a decrease in fitted carrier density does not necessarily imply an equivalent reduction in the total carrier population. Similarly, the localization factor provides phenomenological measures of preferential backscattering but does not directly determine a localization length, defect concentration or universal degree of disorder. Their microscopic origins remain material-dependent, reflecting interlayer and valley effects in MoS_2_, finite size and interparticle transport in N-CND films, nitrogen-related scattering in diamond and irradiation-induced defects in PBN.

Overall, THz magneto-optical spectroscopy provides a versatile framework for examining carrier scattering, preferential backscattering, cyclotron dynamics and effective transport mass across low-dimensional, nanostructured and wide-bandgap materials. Its principal advantage is the ability to obtain complex field-dependent electrodynamic responses without electrical contacts or Kramers–Kronig reconstruction. Quantitative parameters can also be constrained when cyclotron resonance is not spectrally resolved, although their interpretation necessarily depends on the accessible frequency and magnetic-field ranges, sample geometry and selected transport model.

## Figures and Tables

**Table 1:** Neutron irradiation fluences applied to the PBN samples.

Sample	Sample No.	Fluence (*n*·cm^−2^)	Time (Mins)
PBN	A1 A2 A3 A4	0 3.91 × 10^14^ 1.57 × 10^15^ 1.00 × 10^16^	0 13 54 354

**Table 2:** Comparison of the material systems, THz responses, carrier types, cyclotron frequencies and relaxation times at 8 T, and fitted parameter trends. For PBN, the reported ranges correspond to samples A1 and A4. The symbols ↑ and ↓ indicate increasing and decreasing trends with increasing B, respectively; m* denotes the effective carrier mass.

Material System	Sample Geometry	Analyzed Electrodynamic Response	Carrier Type	*ω*_c_ (10^12^ Rads^−1^)	τ (ps)	Fitted Trend with Increasing B
Monolayer MoS_2_ [16]	Atomically thin layer	*σ*_xx_ (*ω*)	Electrons	3.61	0.279	*n*_e_ ↓, *τ* ↑, ∣α∣ ↓, m* fixed
Bilayer MoS_2_ [17]	Atomically thin bilayer	*σ*_±_ (*ω*)	Electrons	2.49	0.133	*n*_e_ ↓, *τ* ↑, ∣α∣ ↓, m* ↑
N-CNDs [4]	sandwich structure	*G*_±_ (*ω*)	Electrons	22.3	0.035	γ↓, *τ* ↑, ∣*α*∣ ↓, m* ↑
N-doped diamond [18]	Bulk	*ϵ*_±_ (*ω*), *σ*_±_ (*ω*)	Electrons	8.18	0.022	*n*_e_↓, *τ* ↑, ∣*α*∣ ↓, m* ↑, *ϵ*_b_ ↑
Neutron-irradiated PBN [5]	Bulk	*ϵ*_±_ (*ω*), *σ*_±_ (*ω*)	Holes	2.88–2.97	0.087–0.099	*n* ↓, *τ* ↑, ∣*α*∣ ↓, m* ↑, *ϵ*_b_ ↑

## Data Availability

No new data were created or analyzed in this study. Data sharing is not applicable to this article.

## References

[1] Ulbricht R., Hendry E., Shan J., Heinz T.F., Bonn M. (2011). Carrier dynamics in semiconductors studied with time-resolved terahertz spectroscopy. Rev. Mod. Phys..

[2] Mei H., Xu W., Wang C., Yuan H., Zhang C., Ding L., Zhang J., Deng C., Wang Y., Peeters F.M. (2018). Terahertz magneto-optical properties of bi-and tri-layer graphene. J. Phys. Condens. Matter.

[3] Mitra S., Avazpour L., Knezevic I. (2025). Terahertz conductivity of two-dimensional materials: A review. J. Phys. Condens. Matter.

[4] Farooq A., Li Y., Xu W., Zhao Z., Wen H., Cheng X., Tang J., Hamdalnile A.A.A., Zhang J., Peeters F.M. (2016). Terahertz magneto-optical properties of nitrogen-doped carbon nanodots on a sapphire substrate. Phys. Rev. B.

[5] Farooq A., Xu W., Wang Z., Cheng X., Hamdalnile A.A.A., Liu L., He G., Peeters F.M. (2025). Electronic properties of neutron-irradiated hexagonal boron nitride measured by terahertz magneto-optical experiment. Infrared Phys. Technol..

[6] Jepsen P.U., Cooke D.G., Koch M. (2011). Terahertz spectroscopy and imaging–Modern techniques and applications. Laser Photonics Rev..

[7] Nagatsuma T. (2011). Terahertz technologies: Present and future. IEICE Electron. Express.

[8] Shi L.-F., Zahid A., Ren A., Ali M.Z., Yue H., Imran M.A., Shi Y., Abbasi Q.H. (2023). The perspectives and trends of THz technology in material research for future communication-a comprehensive review. Physica Scr..

[9] Neu J., Schmuttenmaer C.A. (2018). Tutorial: An introduction to terahertz time domain spectroscopy (THz-TDS). J. Appl. Phys..

[10] Han F., Xu W., Li L., Zhang C. (2016). A generalization of the Drude-Smith formula for magneto-optical conductivities in Faraday geometry. J. Appl. Phys..

[11] Zou X., Shang J., Leaw J., Luo Z., Luo L., La-o-Vorakiat C., Cheng L., Cheong S., Su H., Zhu J.-X. (2013). Terahertz conductivity of twisted bilayer graphene. Phys. Rev. Lett..

[12] Smith N. (2001). Classical generalization of the Drude formula for the optical conductivity. Phys. Rev. B.

[13] Knight S., Hofmann T., Bouhafs C., Armakavicius N., Kühne P., Stanishev V., Ivanov I.G., Yakimova R., Wimer S., Schubert M. (2017). In-situ terahertz optical Hall effect measurements of ambient effects on free charge carrier properties of epitaxial graphene. Sci. Rep..

[14] Poumirol J.-M., Liu P.Q., Slipchenko T.M., Nikitin A.Y., Martin-Moreno L., Faist J., Kuzmenko A.B. (2017). Electrically controlled terahertz magneto-optical phenomena in continuous and patterned graphene. Nat. Commun..

[15] Mu Q., Fan F., Chen S., Xu S., Xiong C., Zhang X., Wang X., Chang S. (2019). Tunable magneto-optical polarization device for terahertz waves based on InSb and its plasmonic structure. Photonics Res..

[16] Wen H., Xu W., Wang C., Song D., Mei H., Zhang J., Ding L. (2021). Magneto-optical properties of monolayer MoS_2_-SiO_2_/Si structure measured via terahertz time-domain spectroscopy. Nano Sel..

[17] Cheng X., Xu W., Wen H., Zhang J., Zhang H., Li H., Peeters F.M. (2024). Key electronic parameters of 2 H-stacking bilayer MoS_2_ on sapphire substrate determined by terahertz magneto-optical measurement in Faraday geometry. Front. Phys..

[18] Xiao H., Wen H., Xu W., Cheng Y., Zhang J., Cheng X., Xiao Y., Ding L., Li H., He B. (2024). Terahertz magneto-optical properties of Nitrogen-doped diamond. Infrared Phys. Technol..

[19] Tao Z., Dong H., Milošević M., Peeters F., Van Duppen B. (2021). Tailoring Dirac plasmons via anisotropic dielectric environment by design. Phys. Rev. Appl..

[20] Tao Z., Lavor I.R., Dong H., Chaves A., Neilson D., Milosevic M.V. (2024). Chiral propagation of plasmon polaritons due to competing anisotropies in a twisted photonic heterostructure. Nano Lett..

[21] Morikawa O., Quema A., Nashima S., Sumikura H., Nagashima T., Hangyo M. (2006). Faraday ellipticity and Faraday rotation of a doped-silicon wafer studied by terahertz time-domain spectroscopy. J. Appl. Phys..

[22] Tinkham M. (1956). Energy gap interpretation of experiments on infrared transmission through superconducting films. Phys. Rev..

[23] Wang C., Xu W., Mei H., Qin H., Zhao X., Zhang C., Yuan H., Zhang J., Xu Y., Li P. (2019). Substrate-induced electronic localization in monolayer MoS_2_ measured via terahertz spectroscopy. Opt. Lett..

[24] Farooq A., Xu W., Zhang J., Wen H., Wang Q., Cheng X., Xiao Y., Ding L., Hamdalnile A.A.A., Li H. (2025). Terahertz optoelectronic properties of monolayer MoS_2_ in the presence of CW laser pumping. Physics.

[25] Song D., Tian J., Xu W., Wen H., Wang C., Tang J., Zhang J., Guo M. (2021). Optically induced insulator-to-semiconductor transition in fluorescent carbon quantum dots measured by terahertz time-domain spectroscopy. Carbon.

[26] Duvillaret L., Garet F., Coutaz J.-L. (1996). A reliable method for extraction of material parameters in terahertz time-domain spectroscopy. IEEE J. Sel. Top. Quantum Electron..

[27] O’Connell R., Wallace G. (1982). Ellipticity and Faraday rotation due to a two-dimensional electron gas in a metal-oxide-semiconductor system. Phys. Rev. B.

[28] Arikawa T., Wang X., Belyanin A.A., Kono J. (2012). Giant tunable Faraday effect in a semiconductor magneto-plasma for broadband terahertz polarization optics. Opt. Express.

[29] Němec H., Kužel P., Sundström V. (2009). Far-infrared response of free charge carriers localized in semiconductor nanoparticles. Phys. Rev. B.

[30] Man L., Xu W., Xiao Y., Wen H., Ding L., Van Duppen B., Peeters F. (2021). Terahertz magneto-optical properties of graphene hydrodynamic electron liquid. Phys. Rev. B.

[31] Manzeli S., Ovchinnikov D., Pasquier D., Yazyev O.V., Kis A. (2017). 2D transition metal dichalcogenides. Nat. Rev. Mater..

[32] Liu P., Xiang B. (2017). 2D hetero-structures based on transition metal dichalcogenides: Fabrication, properties and applications. Sci. Bull..

[33] Mak K.F., Xiao D., Shan J. (2018). Light–valley interactions in 2D semiconductors. Nat. Photonics.

[34] Rose F., Goerbig M., Piéchon F. (2013). Spin-and valley-dependent magneto-optical properties of MoS_2_. Phys. Rev. B.

[35] Kormányos A., Zólyomi V., Drummond N.D., Burkard G. (2014). Spin-orbit coupling, quantum dots, and qubits in monolayer transition metal dichalcogenides. Phys. Rev. X.

[36] Cheiwchanchamnangij T., Lambrecht W.R. (2012). Quasiparticle band structure calculation of monolayer, bilayer, and bulk MoS_2_. Phys. Rev. B.

[37] Tong Q., Yu H., Zhu Q., Wang Y., Xu X., Yao W. (2017). Topological mosaics in moiré superlattices of van der Waals heterobilayers. Nat. Phys..

[38] Kormányos A., Zólyomi V., Fal’ko V.I., Burkard G. (2018). Tunable Berry curvature and valley and spin Hall effect in bilayer MoS_2_. Phys. Rev. B.

[39] Yu Y., Li C., Liu Y., Su L., Zhang Y., Cao L. (2013). Controlled scalable synthesis of uniform, high-quality monolayer and few-layer MoS2 films. Sci. Rep..

[40] Lee Y.-H., Zhang X.-Q., Zhang W., Chang M.-T., Lin C.-T., Chang K.-D., Yu Y.-C., Wang J.T.-W., Chang C.-S., Li L.-J. (2012). Synthesis of large-area MoS_2_ atomic layers with chemical vapor deposition. Adv. Mater..

[41] Mak K.F., Lee C., Hone J., Shan J., Heinz T.F. (2010). Atomically thin MoS_2_: A new direct-gap semiconductor. Phys. Rev. Lett..

[42] Lee Y.-H., Yu L., Wang H., Fang W., Ling X., Shi Y., Lin C.-T., Huang J.-K., Chang M.-T., Chang C.-S. (2013). Synthesis and transfer of single-layer transition metal disulfides on diverse surfaces. Nano Lett..

[43] Wu M.-T., Fan J.-W., Chen K.-T., Chang S.-T., Lin C.-Y. (2015). Band structure and effective mass in monolayer MoS_2_. J. Nanosci. Nanotechnol..

[44] Han C., Noh M., Park H., Yoon J., Cho H., Moon G., Min S.Y., Lee S., Chang H., Choi H. (2025). Terahertz Non-Drude Conductivity of Mirror Twin Boundary Networks on Monolayer MoS_2_ Bicrystals. Nano Lett..

[45] Cheng X., Xu W., Wen H., Zhang J., Zhang H., Li H., Peeters F.M., Chen Q. (2023). Electronic properties of 2H-stacking bilayer MoS_2_ measured by terahertz time-domain spectroscopy. Front. Phys..

[46] Van Der Zande A.M., Kunstmann J., Chernikov A., Chenet D.A., You Y., Zhang X., Huang P.Y., Berkelbach T.C., Wang L., Zhang F. (2014). Tailoring the electronic structure in bilayer molybdenum disulfide via interlayer twist. Nano Lett..

[47] Spitzer W., Fan H. (1957). Determination of optical constants and carrier effective mass of semiconductors. Phys. Rev..

[48] Peeters F., Xiaoguang W., Devreese J. (1986). Cyclotron mass of a polaron in two dimensions. Phys. Rev. B.

[49] Shanmugam M., Durcan C.A., Yu B. (2012). Layered semiconductor molybdenum disulfide nanomembrane based Schottky-barrier solar cells. Nanoscale.

[50] Tang J., Zhang J., Zhang Y., Xiao Y., Shi Y., Chen Y., Ding L., Xu W. (2019). Influence of group modification at the edges of carbon quantum dots on fluorescent emission. Nanoscale Res. Lett..

[51] Chen F., Pei H., Jia Q., Guo W., Zhang X., Guo R., Liu N., Mo Z. (2021). Construction of cyclodextrin functionalized nitrogen-doped graphene quantum dot electrochemical sensing interface for recognition of tryptophan isomers. Mater. Chem. Phys..

[52] Rooj B., Mandal U. (2023). A review on characterization of carbon quantum dots. Vietnam J. Chem..

[53] Tang J., Zhang J., Zhang W., Xiao Y., Shi Y., Kong F., Xu W. (2021). Modulation of red-light emission from carbon quantum dots in acid-based environment and the detection of chromium (III) ions. J. Mater. Sci. Technol..

[54] Ru Y., Sui L., Song H., Liu X., Tang Z., Zang S.Q., Yang B., Lu S. (2021). Rational design of multicolor-emitting chiral carbonized polymer dots for full-color and white circularly polarized luminescence. Angew. Chem. Int. Ed..

[55] Song S., Kim H., Kang C., Bae J. (2023). Terahertz Optical Properties and Carrier Behaviors of Graphene Oxide Quantum Dot and Reduced Graphene Oxide Quantum Dot via Terahertz Time-Domain Spectroscopy. Nanomaterials.

[56] Xu W. (1994). Electron density in Al_x_Ga_1−x_ As/GaAs-based two-dimensional systems in the quantum Hall regime. Phys. Rev. B.

[57] Lax B., Zeiger H. (1957). Possible interpretation of cyclotron resonance absorption in graphite. Phys. Rev..

[58] Hodby J., Russell G., Peeters F., Devreese J., Larsen D. (1987). Cyclotron resonance of polarons in the silver halides: AgBr and AgCl. Phys. Rev. Lett..

[59] Zhang Z., Savukov I., Hilty C. (2025). Large Faraday rotation in pyrolysis synthesized carbon dots. Carbon.

[60] Zhang J., Xu W., Wen H., Cheng X., Zhou S., Li H., Wang Z., He G. (2023). Influence of neutron irradiation on the electronic properties of hexagonal boron nitride measured by terahertz time-domain spectroscopy. Opt. Lett..

[61] Angus J.C., Hayman C.C. (1988). Low-pressure, metastable growth of diamond and “diamondlike” phases. Science.

[62] Tiwari S.K., Pandey R., Wang N., Kumar V., Sunday O.J., Bystrzejewski M., Zhu Y., Mishra Y.K. (2022). Progress in diamanes and diamanoids nanosystems for emerging technologies. Adv. Sci..

[63] Ejalonibu H., Sarty G., Bradley M. (2020). The effect of step-wise surface nitrogen doping in MPECVD grown polycrystalline diamonds. Mater. Sci. Eng. B.

[64] Zhao Y., Li C., Liu J., An K., Yan X., Hei L., Chen L., Wei J., Lu F. (2019). The interface and mechanical properties of a CVD single crystal diamond produced by multilayered nitrogen doping epitaxial growth. Materials.

[65] Wang Y., Zhang W., Lin C.-N., Ren P., Lu Y.-J., Shan C., Yu S.F. (2018). Multiphoton upconversion emission from diamond single crystals. ACS Appl. Mater. Interfaces.

[66] Ficek M., Sankaran K.J., Ryl J., Bogdanowicz R., Lin I.-N., Haenen K., Darowicki K. (2016). Ellipsometric investigation of nitrogen doped diamond thin films grown in microwave CH4/H2/N2 plasma enhanced chemical vapor deposition. Appl. Phys. Lett..

[67] Ulbricht R., van der Post S.T., Goss J.P., Briddon P.R., Jones R., Khan R.U.A., Bonn M. (2011). Single substitutional nitrogen defects revealed as electron acceptor states in diamond using ultrafast spectroscopy. Phys. Rev. B.

[68] Xiao H., Wen H., Xu W., Zhang J., Cheng X.-J., Xiao Y.-M., Ding L., Li H.-W., Cheng Y.-Z., He B. (2023). Faraday rotation in nitrogen-doped diamond measured by polarized terahertz time-domain spectroscopy in the presence of strong magnetic field. J. Infrared Millim. Waves.

[69] Xiao H., Zhang Z., Xu W., Wang Q., Xiao Y., Ding L., Huang J., Li H., He B., Peeters F.M. (2023). Terahertz optoelectronic properties of synthetic single crystal diamond. Diam. Relat. Mater..

[70] Aoki H., Ando T. (1981). Effect of localization on the Hall conductivity in the two-dimensional system in strong magnetic fields. Solid State Commun..

[71] Redman D., Shu Q., Brown S., Lenef A., Lrj Y., Whitaker J., Rand S., Satoh S., Tsuji K., Yazu S. (1992). Electronic structure of NV centers and terahertz spectroscopy of diamond. MRS Online Proc. Libr. (OPL).

[72] De Leeuw S., Perram J.W., Smith E. (1986). Computer simulation of the static dielectric constant of systems with permanent electric dipoles. Annu. Rev. Phys. Chem..

[73] Löfås H., Grigoriev A., Isberg J., Ahuja R. (2011). Effective masses and electronic structure of diamond including electron correlation effects in first principles calculations using the GW-approximation. AIP Adv..

[74] Naka N., Fukai K., Handa Y., Akimoto I. (2013). Direct measurement via cyclotron resonance of the carrier effective masses in pristine diamond. Phys. Rev. B.

[75] Caldwell J.D., Aharonovich I., Cassabois G., Edgar J.H., Gil B., Basov D.N. (2019). Photonics with hexagonal boron nitride. Nat. Rev. Mater..

[76] Duan X., Jia D., Wang Z., Cai D., Tian Z., Yang Z., He P., Wang S., Zhou Y. (2016). Influence of hot-press sintering parameters on microstructures and mechanical properties of h-BN ceramics. J. Alloys Compd..

[77] Zhou S., Xu W., Xiao Y., Xiao H., Zhang J., Wang Z., He G., Liu J., Li Y., Peeters F.M. (2023). Influence of neutron irradiation on X-ray diffraction, Raman spectrum and photoluminescence from pyrolytic and hot-pressed hexagonal boron nitride. J. Lumin..

[78] Li J., Glaser E.R., Elias C., Ye G., Evans D., Xue L., Liu S., Cassabois G., Gil B., Valvin P. (2021). Defect engineering of monoisotopic hexagonal boron nitride crystals via neutron transmutation doping. Chem. Mater..

[79] Buyuk B., Goncu Y., Tugrul A.B., Ay N. (2020). Swelling on neutron induced hexagonal boron nitride and hexagonal boron nitride-titanium diboride composites. Vacuum.

[80] Toledo J.R., de Jesus D.B., Kianinia M., Leal A.S., Fantini C., Cury L.A., Sáfar G.A.M., Aharonovich I., Krambrock K. (2018). Electron paramagnetic resonance signature of point defects in neutron-irradiated hexagonal boron nitride. Phys. Rev. B.

[81] Li J., Dahal R., Majety S., Lin J., Jiang H. (2011). Hexagonal boron nitride epitaxial layers as neutron detector materials. Nucl. Instrum. Methods Phys. Res. Sect. A Accel. Spectrom. Detect. Assoc. Equip..

[82] Jiang H., Lin J.Y. (2017). Review—Hexagonal boron nitride epilayers: Growth, optical properties and device applications. ECS J. Solid State Sci. Technol..

[83] Konusov F.V., Kabyshev A.V., Pavlov S.K., Tarbokov V.A., Remnev G.E. (2019). Effect of short-pulsed ion irradiation on the optical and electrical properties of pyrolytic boron nitride. Nucl. Instrum. Methods Phys. Res. Sect. B Beam Interact. Mater. At..

[84] Huber A.J., Keilmann F., Wittborn J., Aizpurua J., Hillenbrand R. (2008). Terahertz near-field nanoscopy of mobile carriers in single semiconductor nanodevices. Nano Lett..

[85] Wang J., Rickel D.G., Chang C., Zhang Z., Peng P., Huang Y., Azad A.K., Jena D., Xing H.G., Crooker S.A. (2025). THz cyclotron resonance of a 2D hole gas in a GaN/AlN heterostructure. Appl. Phys. Lett..

[86] Shimano R., Yumoto G., Yoo J.Y., Matsunaga R., Tanabe S., Hibino H., Morimoto T., Aoki H. (2013). Quantum Faraday and Kerr rotations in graphene. Nat. Commun..

[87] Wu L., Salehi M., Koirala N., Moon J., Oh S., Armitage N. (2016). Quantized Faraday and Kerr rotation and axion electrodynamics of a 3D topological insulator. Science.

[88] Dastrup B.S., Miedaner P.R., Zhang Z., Nelson K.A. (2024). Optical-pump–terahertz-probe spectroscopy in high magnetic fields with kHz single-shot detection. Rev. Sci. Instrum..

[89] Wang L., Shih E.-M., Ghiotto A., Xian L., Rhodes D.A., Tan C., Claassen M., Kennes D.M., Bai Y., Kim B. (2020). Correlated electronic phases in twisted bilayer transition metal dichalcogenides. Nat. Mater..

[90] Lloyd D., Liu X., Christopher J.W., Cantley L., Wadehra A., Kim B.L., Goldberg B.B., Swan A.K., Bunch J.S. (2016). Band Gap Engineering with Ultralarge Biaxial Strains in Suspended Monolayer MoS_2_. Nano Lett..

[91] Cocker T.L., Baillie D., Buruma M., Titova L.V., Sydora R.D., Marsiglio F., Hegmann F.A. (2017). Microscopic origin of the Drude-Smith model. Phys. Rev. B.

[92] Maity A., Grenadier S.J., Li J., Lin J.Y., Jiang H.X. (2018). Hexagonal boron nitride neutron detectors with high detection efficiencies. J. Appl. Phys..

[93] Crassee I., Levallois J., Walter A.L., Ostler M., Bostwick A., Rotenberg E., Seyller T., van der Marel D., Kuzmenko A.B. (2011). Giant Faraday rotation in single- and multilayer graphene. Nat. Phys..

